# Associations Between Radiation Oncologist Demographic Factors and Segmentation Similarity Benchmarks: Insights From a Crowd-Sourced Challenge Using Bayesian Estimation

**DOI:** 10.1200/CCI.23.00174

**Published:** 2024-06-13

**Authors:** Kareem A. Wahid, Onur Sahin, Suprateek Kundu, Diana Lin, Anthony Alanis, Salik Tehami, Serageldin Kamel, Simon Duke, Michael V. Sherer, Mathis Rasmussen, Stine Korreman, David Fuentes, Michael Cislo, Benjamin E. Nelms, John P. Christodouleas, James D. Murphy, Abdallah S.R. Mohamed, Renjie He, Mohammed A. Naser, Erin F. Gillespie, Clifton D. Fuller

**Affiliations:** 1 Department of Radiation Oncology, The University of Texas MD Anderson Cancer Center, Houston, TX; 2 Department of Imaging Physics, The University of Texas MD Anderson Cancer Center, Houston, TX; 3 Department of Biostatistics, The University of Texas MD Anderson Cancer Center, Houston, TX; 4 Department of Radiation Oncology, Memorial Sloan Kettering Cancer Center, New York, NY; 5 Department of Radiation Oncology, Cambridge University Hospitals, Cambridge, United Kingdom; 6 Department of Radiation Medicine and Applied Sciences, University of California San Diego, La Jolla, CA; 7 Department of Oncology, Aarhus University Hospital, Aarhus, Denmark; 8 Canis Lupus, LLC, Merrimac, WI; 9 Department of Radiation Oncology, The University of Pennsylvania Cancer Center, Philadelphia, PA; 10 Elekta, Atlanta, GA; 11 Department of Radiation Oncology, University of Washington Fred Hutchinson Cancer Center, Seattle, WA

## Abstract

**PURPOSE:**

The quality of radiotherapy auto-segmentation training data, primarily derived from clinician observers, is of utmost importance. However, the factors influencing the quality of clinician-derived segmentations are poorly understood; our study aims to quantify these factors.

**METHODS:**

Organ at risk (OAR) and tumor-related segmentations provided by radiation oncologists from the Contouring Collaborative for Consensus in Radiation Oncology data set were used. Segmentations were derived from five disease sites: breast, sarcoma, head and neck (H&N), gynecologic (GYN), and GI. Segmentation quality was determined on a structure-by-structure basis by comparing the observer segmentations with an expert-derived consensus, which served as a reference standard benchmark. The Dice similarity coefficient (DSC) was primarily used as a metric for the comparisons. DSC was stratified into binary groups on the basis of structure-specific expert-derived interobserver variability (IOV) cutoffs. Generalized linear mixed-effects models using Bayesian estimation were used to investigate the association between demographic variables and the binarized DSC for each disease site. Variables with a highest density interval excluding zero were considered to substantially affect the outcome measure.

**RESULTS:**

Five hundred seventy-four, 110, 452, 112, and 48 segmentations were used for the breast, sarcoma, H&N, GYN, and GI cases, respectively. The median percentage of segmentations that crossed the expert DSC IOV cutoff when stratified by structure type was 55% and 31% for OARs and tumors, respectively. Regression analysis revealed that the structure being tumor-related had a substantial negative impact on binarized DSC for the breast, sarcoma, H&N, and GI cases. There were no recurring relationships between segmentation quality and demographic variables across the cases, with most variables demonstrating large standard deviations.

**CONCLUSION:**

Our study highlights substantial uncertainty surrounding conventionally presumed factors influencing segmentation quality relative to benchmarks.

## Teaser

Common radiation oncologist demographic factors are not associated with segmentation performance.

## INTRODUCTION

Segmentation (also termed contouring) of regions of interest (ROIs) on medical images is crucial for radiotherapy planning.^1^ Importantly, accurate segmentation of organs at risk (OARs) and tumor-related (ie, target) structures is required to optimize radiotherapeutic efficacy. Segmentation is often performed by clinicians, such as radiation oncologists. However, clinician-derived manual segmentation is a time- and labor-intensive task, thereby prompting the increasing development of artificial intelligence (AI)–based methods for auto-segmentation.^2^

**CONTEXT**

**Key Objective**
How do demographic factors of radiation oncologists influence the quality of radiotherapy-related segmentation in medical imaging?
**Knowledge Generated**
Tumor-related structures were generally more challenging to segment than organs at risk. However, the impact of demographic factors on segmentation quality was minimal and characterized by high uncertainty.
**Relevance *(J.L. Warner)***
This study further provides guidance regarding how to best implement autosegmentation within clinical practice. Specifically, it suggests that tumor structures should likley be auto-segmented with caution.*
*Relevance section written by *JCO Clinical Cancer Informatics* Editor-in-Chief Jeremy L. Warner, MD, MS, FAMIA, FASCO.

The Contouring Collaborative for Consensus in Radiation Oncology (C3RO), a large-scale crowdsourcing challenge for radiotherapy segmentation, demonstrated that nonexpert consensus segmentations could quantitatively approximate expert consensus segmentations in a variety of disease sites,^3^ thereby motivating the potential use of a large number of lower-quality segmentations in place of a small number of high-quality segmentations for AI model training. Notably, segmentations were highly variable among the participants of C3RO, suggesting underlying factors associated with resultant segmentation quality.

Despite AI advancements, human clinicians will likely be involved in the radiotherapy segmentation process for the foreseeable future, both as suppliers of ground truth for algorithmic training and as the final arbiters of quality. Understanding the characteristics of clinicians associated with superior segmentation performance could help guide training, inform the design of auto-segmentation tools, and ultimately improve the quality of care provided to patients. While some data do suggest that clinician experience is associated with improved radiotherapy outcomes,^4-6^ no studies have directly examined underlying factors related to segmentation quality. Therefore, we aim to investigate whether demographic factors of a large number of radiation oncologists are associated with improved segmentation quality through a secondary analysis of C3RO.

## METHODS

### Study Participants and Demographic Variables

Participants in C3RO were categorized as recognized experts or nonexperts. Recognized experts were identified by the C3RO organizers as board-certified physicians who participated in the development of national guidelines and/or contributed to extensive scholarly activities within a specific disease site. Nonexperts were any participants not categorized as an expert for that disease site. For this study, nonexpert participants from each separate disease site of the C3RO database, namely, the breast, sarcoma, head and neck (H&N), gynecologic (GYN), and GI cases, were selected for the analysis. Greater details on the publicly available C3RO data set can be found in the corresponding data descriptor.^7^ Self-reported demographic variables of interest from the participants were initially collected through an intake survey performed on REDCap.^8^ Informed by previous research,^9,10^ various demographic variables were collected for physicians in this study (Table 1). Before use in the analysis, nonexpert participants were filtered out of the data set if they were trainees (eg, residents) or nonphysicians (eg, radiation therapists, medical physicists, other). The primary practice description variable was converted to a binary format by grouping academic/university (academic) into one group and all others into a separate group (nonacademic).

**TABLE 1.** Demographic Variables Examined in This Study

Variable	Description
Practice location	Geographic location where participant actively practices. Binary variable with possible values of US or non-US
Sex	Self-identified sex. Binary variable with possible values of male or female. Original variable included nonbinary as an option but was not selected by any participants
Race	Self-identified race. Binary variable with possible values of White or non-White
Academic affiliation	Whether the participant actively holds an academic affiliation. Binary variable with possible values of yes or no
Primary practice type	Best self-identified description of primary practice where the participant actively practices. Categorical variables with values of academic/university, nonacademic hospital, private practice (solo or group), or other. Converted to a binary variable with possible values of academic or nonacademic
No. of radiation oncologist colleagues	The total estimated number of radiation oncologist colleagues who work with the participant at their primary clinical site (excluding themselves). Continuous numerical variable with a minimum value of 0
Presence of another radiation oncologist during clinic	Whether there is at least one additional radiation oncologist when the participant is actively working in their clinic (on most days). Binary variable with possible values of yes or no
Actively treat disease site	Whether the participant actively treats the disease site under investigation, for example, for the breast case, do they actively treat breast patients in their clinic? Binary variable with possible values of yes or no
Years of practice	No. of self-reported years since completing residency (calculated relative to the start of C3RO). Continuous numerical variable with a minimum value of 0

Abbreviation: C3RO, Contouring Collaborative for Consensus in Radiation Oncology.

### Segmentation Evaluation

**FIG 1.**
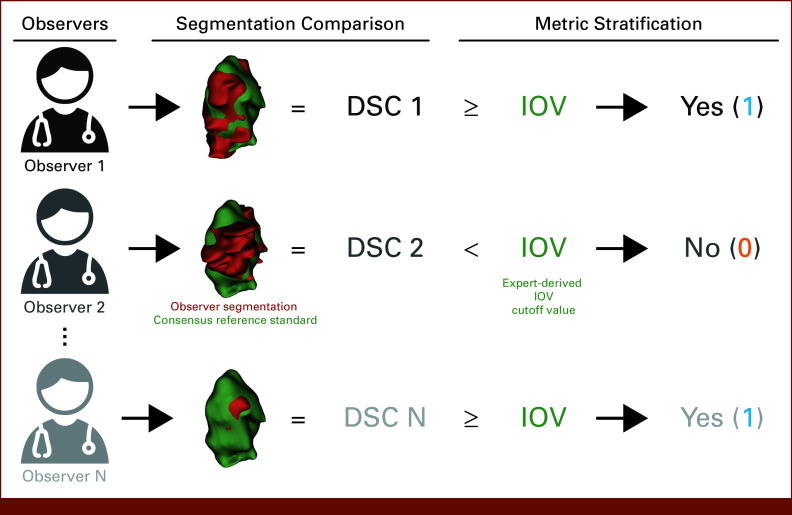
Derivation of binarized structure segmentation quality for each observer. Each observer could segment multiple structures, that is, organs at risk and tumor volumes. Observer segmentations (red volume) were compared with a reference standard derived from a consensus segmentation of experts (green volume) using the DSC. Segmentation metrics were then stratified into being greater than or equal to (yes—1) or below (no—0) a previously derived expert-derived IOV cutoff value for that particular region of interest. In this example, the primary gross tumor volume structure for the head and neck case is shown. A similar process was used to derive binarized values for surface DSC. DSC, Dice similarity coefficient; IOV, interobserver variability.

All ROIs from all disease sites in the C3RO data set were used for this analysis (Data Supplement, Table S1). Notably, participants generated ROI segmentations based principally on contrast-enhanced radiotherapy planning computed tomography scans. Participants were provided a short clinical history for each case. Additional case-specific considerations included the following: the breast case not receiving contrast, the H&N and GI cases having positron emission tomography scans available for reference, and the sarcoma case having a magnetic resonance imaging scan available for reference. For each nonexpert ROI, we calculated segmentation quality by comparing the nonexpert segmentation with the consensus of experts as derived using the Simultaneous Truth and Performance Level Estimation (STAPLE) algorithm^11^ (Fig 1). The number of expert observers used for each ROI consensus segmentation is presented in the Data Supplement (Table S1). It should be noted that as with any segmentation study, there was no definitive underlying ground truth set of segmentations we could reference. Although experts were subjectively determined in the original C3RO study, they demonstrated significantly improved interobserver variability (IOV) compared with their nonexpert counterparts.^3^ Therefore, the expert STAPLE can be considered as a reference standard segmentation. We used the existing Neuroimaging Informatics Technology Initiative structure files for comparisons, which were previously converted from Digital Imaging and Communications in Medicine using DICOMRTTool.^12^ The Dice similarity coefficient (DSC) was used as the main metric for comparison because of its ubiquity.^1^ We also investigated two metrics of surface similarity, the surface DSC (SDSC) and 95% Hausdorff distance (HD95), for additional experiments; SDSC tolerance values for each ROI were determined from the pairwise average surface distance of the expert segmentations. Metrics were calculated using the surface-distance Python package v. 0.1^13^ and in-house Python code (Python v. 3.11.4).

To ensure that metrics were comparable across ROIs, metrics were stratified into binary groups on the basis of previously established ROI-specific expert-derived IOV cutoffs—cutoffs were calculated as the median of pairwise metric values for all available expert segmentations.^7^ Namely, if the metric for a given ROI was greater than or equal to the ROI-specific expert IOV, it was classified as 1, otherwise, 0 (Fig 1). Finally, for each ROI, we calculated the percentage of observers who were able to cross the expert IOV cutoff.

### Bayesian Regression Analysis

Generalized linear mixed-effects models with Bayesian estimation were used to investigate the relationship between demographic variables and binarized segmentation quality metrics. The stratified binary segmentation quality metric acted as the dependent variable for the models. The key independent variables were practice location, primary practice type, number of radiation oncologist colleagues, presence of another radiation oncologist during clinic, actively treat disease site, and years of practice. Notably, exploratory correlative analysis (Data Supplement, Figs S1-S5) revealed high relative correlation between academic affiliation and primary practice type; therefore, academic affiliation was not included as a covariate to facilitate model parsimony. An additional binary categorical variable, ROI type, was added as an independent variable to indicate if the ROI was an OAR or tumor volume. Furthermore, models were corrected for self-identified sex and self-identified race by including them as independent variables in the models. A random intercept was used in the models to account for the various observers who could segment multiple structures on the same image. Any empty values for numerical variables were imputed to the median value relative to the total number of observations. Finally, numerical variables were Z-score normalized.

The Python package Bambi v. 0.12.0,^14^ which is built on top of the robust Markov chain Monte Carlo (MCMC) library PyMC3,^15^ was used for all regression analyses. For each disease site, the regression formula was defined as

$$Y_{ij}\;∼\;\text{Bernoulli}\left(p_{ij}\right),$$

$$\text{logit}\left(p_{ij}\right)=\beta 0+u_{j}+\beta 1\times \left(\text{Locatio}n_{ij}\right)+\beta 2\times \left(\text{Practic}{e\_\text{type}}_{ij}\right)+\beta 3\times \left(\text{Number}\;\text{of}\;\text{Colleague}s_{ij}\right)+\beta 4\times \left(\text{Colleagu}{e\_\text{presence}}_{ij}\right)+\beta 5\times \left(\text{Trea}{t\_\text{site}}_{ij}\right)+\beta 6\times \left(\text{Number}\;\text{of}\;\text{Year}{s\_\text{practice}}_{ij}\right)+\beta 7\times \left(\text{ROI}\_\text{typ}e_{ij}\right)+\beta 8\times \left(\text{Gende}r_{ij}\right)+\beta 9\times \left(\text{Rac}e_{ij}\right)$$,

where $Y_{ij}$ is the dependent variable for observation i nested within observer j, which follows a Bernoulli distribution with success probability $p_{ij}$; $\text{logit}\left(p_{ij}\right)$ is the log odds of the success probability; β0 is the overall intercept; $u_{j}$ is the random intercept for observer j; β1, …, β9 are the fixed effect coefficients for the predictors.

For each MCMC Bayesian regression model, 10,000 samples were drawn from four chains with a tuning set of 1,500 iterations for a total of 46,000 samples drawn for each model. Weakly informative priors as determined using the Bambi package were intelligently generated for all model terms by loosely scaling them to the observed data.^14^ Computations were performed across six cores of an Intel Core i7-8700 Processor.

The ArviZ v. 0.15.1^16^ Python library was used to derive summary data for the posterior distribution. Point estimates (posterior means) and assessments of uncertainty (posterior standard deviation) were calculated for each variable. In addition, the 89% highest density interval (HDI) was calculated; a value of 89% was selected as suggested in recent literature.^17,18^ Demographic variables for which the HDI did not include zero were considered to have a substantial impact on the outcome measure of interest and could be interpreted as loosely analogous to the frequentist notion of statistical significance.

### Data and Code Availability

All C3RO data, including the original demographic factors and segmentation data, are available on Figshare (DOI = doi.org/10.6084/m9.figshare.21074182). All Python code used for this study are available on GitHub.^19^ Corresponding newly created data can also be found on Figshare (DOI = doi.org/10.6084/m9.figshare.24021591).

## RESULTS

### Study Participants

**FIG 2.**
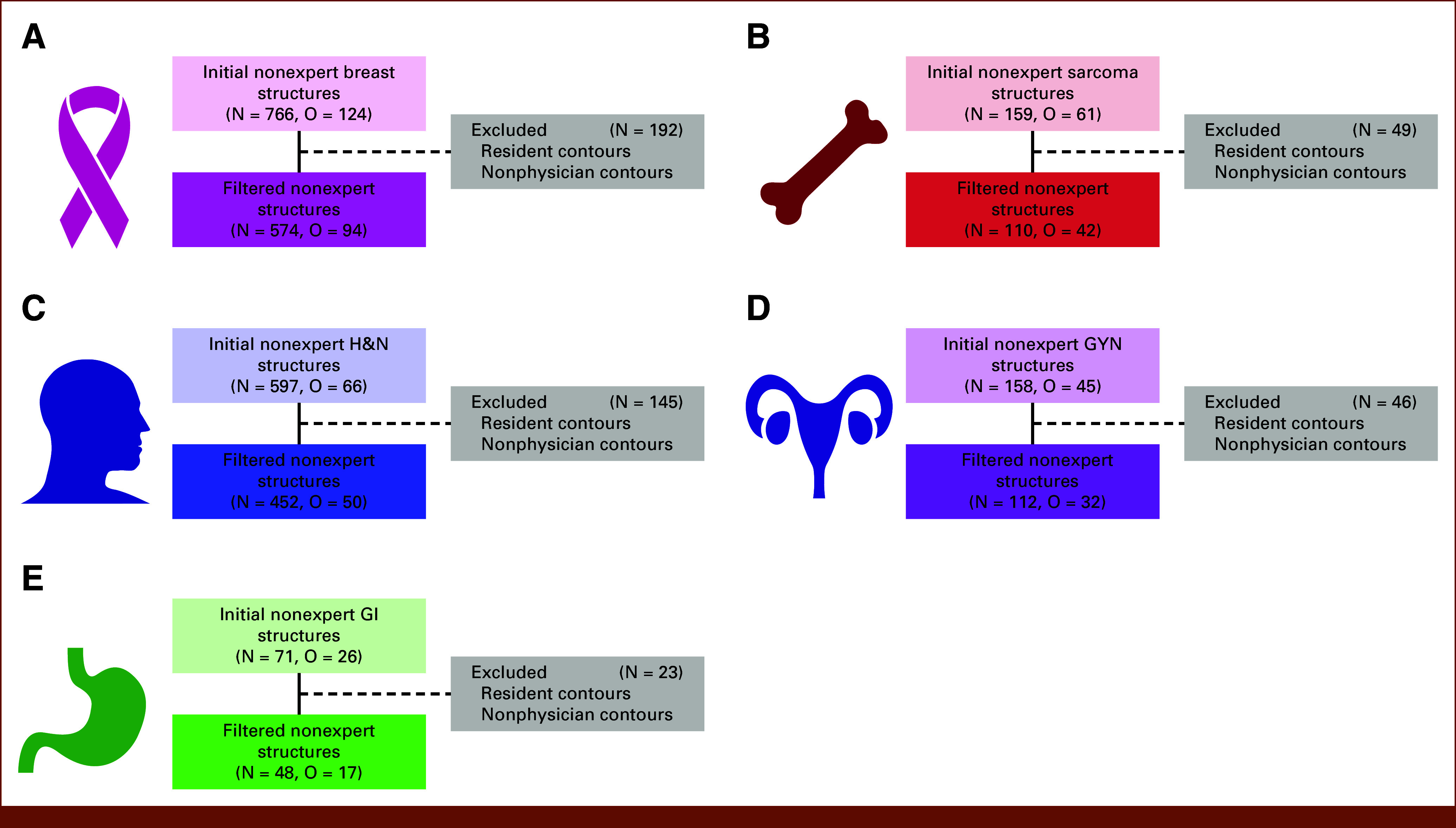
Flow diagrams showing the final number of structure segmentations evaluated for each disease site. Breast, sarcoma, H&N, GYN, and GI cases are shown in panels (A-E), respectively. GYN, gynecologic; H&N, head and neck; N, number of nonexpert structure segmentations; O, number of unique nonexpert observers.

After filtering out structures from noneligible observers, 574, 110, 452, 112, and 48 ROI structure observations from practicing radiation oncologist observers remained for the analysis for the breast, sarcoma, H&N, GYN, and GI cases, respectively (Fig 2). Descriptive statistics for the clinician observers used in our study are presented in the Data Supplement (Tables S2 and S3).

### Individual Observer Performance

**FIG 3.**
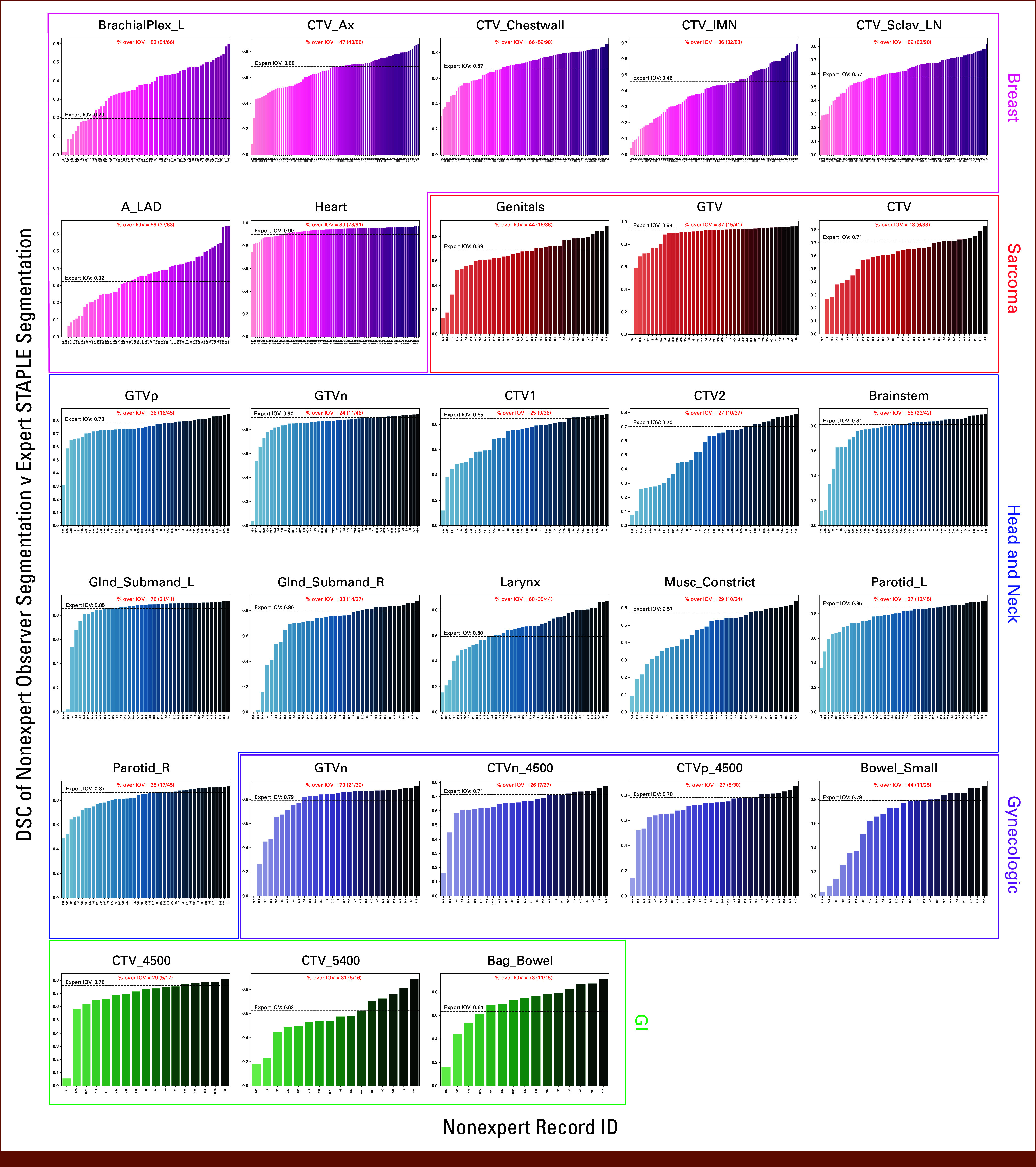
Bar plots of individual observer segmentation performance versus reference standard. Magenta, red, blue, purple, and green plots correspond to breast, sarcoma, head and neck, gynecologic, and GI ROIs, respectively. The reference standard segmentation is the consensus segmentation of all experts as derived from the Simultaneous Truth and Performance Level Estimation algorithm. Black dashed lines indicate median expert interobserver DSC for a corresponding ROI. The percentage of observers that crossed the expert IOV cutoff is shown in red above each plot. DSC, Dice similarity coefficient; IOV, interobserver variability; ROI, region of interest; STAPLE, Simultaneous Truth and Performance Level Estimation.

Figure 3 shows the DSC scores for each observer relative to the expert consensus segmentation stratified by ROI; the percentage of observations that were able to cross the expert IOV cutoff are also shown. The highest percentages per case were BrachialPlex_L (82%), Genitals (44%), Glnd_Submand_L (76%), GTV n (70%), and Bag_Bowel (73%) for breast, sarcoma, H&N, GYN, and GI, respectively. The lowest percentages per case were CTV_IMN (36%), CTV (18%), GTVn (24%), CTVn_4500 (26%), and CTV_4500 (29%) for breast, sarcoma, H&N, GYN, and GI, respectively. Aggregated median percentage values when stratified by ROI type were 55% (IQR, 35%) and 31% (IQR, 15%) for OARs and tumor volumes, respectively. Analogous bar plots using SDSC and HD95 as metrics are shown in the Data Supplement (Figs S6-S15). SDSC and HD95 values mirrored DSC values for most ROIs. Aggregated SDSC median percentage values when stratified by ROI type were 36% (IQR, 32%) and 30% (IQR, 30%) for OARs and tumor volumes, respectively. Aggregated HD95 median percentage values when stratified by ROI type were 57% (IQR, 40%) and 41% (IQR, 26%) for OARs and tumor volumes, respectively.

### Bayesian Regression Models

Mixed-effects regression results using binarized DSC and binarized SDSC as the outcome variables are shown in Tables 2 and 3, respectively. For the breast case, tumor-related ROI type for both DSC (mean ± standard deviation, –0.97 ± 0.20) and SDSC (–1.24 ± 0.20) had HDIs that excluded zero. For the sarcoma case, the tumor-related ROI type for both DSC (–1.04 ± 0.54) and SDSC (–2.74 ± 0.81) had HDIs that excluded zero. For the H&N case, the DSC tumor-related ROI type (–1.00 ± 0.24) and DSC White racial self-identification (0.66 ± 0.41) had HDIs that excluded zero. For the GYN case, only the SDSC academic practice type (–1.30 ± 0.79) had an HDI that excluded zero. For the GI case, the DSC tumor-related ROI type (–2.95 ± 0.98) and DSC colleague presence (2.21 ± 1.40) had HDIs that excluded zero. Additional regression results for HD95 are presented in the Data Supplement (Table S4). Model convergence parameters estimated for each variable are presented in the Data Supplement (Tables S5-S19).

**TABLE 2.** Generalized Linear Mixed-Effects Models With Bayesian Estimation Results Using Binarized Dice Similarity Coefficient as the Outcome Variable

Variable	Breast		Sarcoma		Head and Neck		Gynecologic		GI	
	Mean (HDI)	SD	Mean (HDI)	SD	Mean (HDI)	SD	Mean (HDI)	SD	Mean (HDI)	SD
Intercept	0.90 (0.08-1.69)	0.51	0.76 (–1.30 to 2.93)	1.35	–0.46 (–1.85 to 0.87)	0.86	–0.10 (–2.02 to 1.78)	1.21	–3.28 (–7.34 to 1.08)	2.67
ROI type (tumor)	**–0.97** (**–1.29 to –0.65)**	0.20	**–1.04 (–1.91 to –0.19)**	0.54	**–1.00 (–1.39 to –0.62)**	0.24	–0.09 (–0.92 to 0.72)	0.51	**–2.95 (–4.44 to –1.36)**	0.98
Location (United States)	–0.54 (–1.15 to 0.06)	0.38	0.55 (–1.16 to 2.21)	1.07	0.07 (–0.92 to 1.06)	0.62	0.08 (–1.52 to 1.66)	1.01	4.24 (–0.69 to 9.36)	3.22
Sex (female)	–0.10 (–0.51 to 0.31)	0.26	0.66 (–0.60 to 1.91)	0.80	–0.26 (–1.01 to 0.47)	0.47	–0.13 (–1.15 to 0.88)	0.64	0.55 (–1.25 to 2.35)	1.14
Years of practice	–0.06 (–0.25 to 0.13)	0.12	–0.44 (–1.07 to 0.18)	0.41	–0.23 (–0.56 to 0.11)	0.21	–0.25 (–0.78 to 0.24)	0.33	–0.33 (–1.49 to 0.83)	0.74
Practice type (academic)	–0.37 (–0.74 to 0.02)	0.24	–0.01 (–1.24 to 1.21)	0.79	0.13 (–0.48 to 0.75)	0.39	–0.37 (–1.34 to 0.59)	0.62	–1.37 (–3.87 to 1.12)	1.58
No. of colleagues	–0.02 (–0.22 to 0.18)	0.12	–0.15 (–0.82 to 0.53)	0.43	0.22 (–0.15 to 0.58)	0.23	–0.13 (–0.61 to 0.36)	0.31	0.01 (–1.51 to 1.59)	0.98
Colleague presence (yes)	0.28 (–0.21 to 0.77)	0.31	–1.13 (–2.60 to 0.37)	0.95	0.24 (–0.62 to 1.07)	0.53	0.23 (–1.06 to 1.57)	0.83	**2.21 (0.03-4.43)**	1.40
Race (White)	–0.30 (–0.68 to 0.09)	0.24	–0.77 (–2.06 to 0.53)	0.83	**0.66 (0.02-1.33)**	0.41	0.47 (–0.47 to 1.36)	0.58	0.89 (–0.91 to 2.70)	1.15
Treat disease site (yes)	0.53 (–0.14 to 1.20)	0.42	–0.39 (–1.62 to 0.84)	0.82	–0.13 (–1.33 to 1.02)	0.74	–0.42 (–1.76 to 0.92)	0.85	3.02 (–0.34 to 6.26)	2.09
Random effect variance	0.57 (0.27-0.90)	0.19	1.44 (0.18-2.47)	0.74	1.03 (0.67-1.39)	0.23	0.73 (0.00-1.31)	0.46	0.78 (0.00-1.58)	0.64

NOTE. Model coefficient values are shown for each variable. Reference variables for categorical variables are shown in brackets next to the variable name. Sign value in posterior mean indicates positive or negative correlation of variable with outcome. Posterior SD indicates uncertainty around posterior mean. Eighty-nine percent HDI is shown in parentheses after posterior mean. Variables in bold indicate that HDI does not contain zero and is considered to have a substantial impact on the outcome measure of interest.

Abbreviations: HDI, highest density interval; ROI, regions of interest; SD, standard deviation.

**TABLE 3.** Generalized Linear Mixed-Effects Models With Bayesian Estimation Results Using Binarized Surface Dice Similarity Coefficient as the Outcome Variable

Variable	Breast		Sarcoma		Head and Neck		Gynecologic		GI	
	Mean (HDI)	SD	Mean (HDI)	SD	Mean (HDI)	SD	Mean (HDI)	SD	Mean (HDI)	SD
Intercept	0.70 (–0.13 to 1.50)	0.51	2.30 (–1.17 to 5.80)	2.25	–1.34 (–2.53 to –0.13)	0.76	–0.28 (–2.65 to 2.14)	1.51	2.79 (–1.67 to 7.20)	2.85
ROI type (tumor)	**–1.24** (**–1.54 to –0.91)**	0.20	**–2.74** (**–3.97 to –1.44)**	0.81	–0.37 (–0.77 to 0.00)	0.24	0.56 (–0.38 to 1.46)	0.58	–0.16 (–1.48 to 1.23)	0.86
Location (United States)	–0.33 (–0.95 to 0.25)	0.38	0.93 (–1.77 to 3.66)	1.73	–0.28 (–1.15 to 0.60)	0.55	–0.47 (–2.44 to 1.49)	1.24	0.70 (–3.10 to 4.66)	2.52
Sex (female)	–0.30 (–0.69 to 0.10)	0.25	0.75 (–1.26 to 2.93)	1.33	–0.24 (–0.87 to 0.40)	0.40	–0.06 (–1.34 to 1.27)	0.83	–0.42 (–2.40 to 1.69)	1.30
Years of practice	–0.01 (–0.19 to 0.18)	0.12	0.10 (–0.78 to 0.99)	0.57	–0.25 (–0.55 to 0.03)	0.18	–0.54 (–1.18 to 0.13)	0.42	0.94 (–0.40 to 2.31)	0.86
Practice type (academic)	–0.37 (–0.75 to 0.01)	0.24	–0.53 (–2.58 to 1.48)	1.29	0.40 (–0.15 to 0.92)	0.34	**–1.30** (**–2.52 to –0.04)**	0.79	–1.49 (–4.41 to 1.42)	1.85
No. of colleagues	–0.03 (–0.23 to 0.17)	0.13	–0.06 (–1.17 to 1.07)	0.71	0.09 (–0.22 to 0.40)	0.20	–0.34 (–1.01 to 0.34)	0.43	1.18 (–0.68 to 3.00)	1.17
Colleague presence (yes)	0.16 (–0.34 to 0.63)	0.30	–2.10 (–4.61 to 0.31)	1.58	0.38 (–0.36 to 1.14)	0.47	0.27 (–1.39 to 1.98)	1.07	1.61 (–0.71 to 3.99)	1.53
Race (White)	–0.17 (–0.54 to 0.20)	0.23	–0.81 (–2.95 to 1.32)	1.36	0.22 (–0.34 to 0.78)	0.36	0.65 (–0.50 to 1.80)	0.73	–0.30 (–2.61 to 1.88)	1.45
Treat disease site (yes)	0.67 (–0.01 to 1.34)	0.43	–1.11 (–3.17 to 1.00)	1.33	0.05 (–1.02 to 1.07)	0.66	–0.66 (–2.39 to 1.09)	1.10	–1.75 (–5.09 to 1.35)	2.08
Random effect variance	0.58 (0.29-0.89)	0.19	2.93 (1.11-4.60)	1.14	0.78 (0.42-1.14)	0.23	1.15 (0.08-1.94)	0.59	1.30 (0.00-2.60)	1.06

NOTE. Model coefficient values are shown for each variable. Reference variables for categorical variables are shown in brackets next to the variable name. Sign value in posterior mean indicates positive or negative correlation of variable with outcome. Posterior SD indicates uncertainty around posterior mean. Eighty-nine percent HDI is shown in parentheses after posterior mean. Variables in bold indicate that HDI does not contain zero and is considered to have a substantial impact on the outcome measure of interest.

Abbreviations: HDI, highest density interval; ROI, regions of interest; SD, standard deviation.

## DISCUSSION

To date, there are limited standardized measures to evaluate radiotherapy-related segmentation quality. Nissen et al^20^ recently proposed the utilization of the Jaccard Index for longitudinal quantitative evaluation. However, the inherent quality discerned from these metrics in their raw numerical form often varies on the basis of the specific ROI. For example, a DSC of 0.80 for a particularly simple OAR may be less desirable than a DSC of 0.80 for a particularly difficult tumor volume. However, stratification of evaluation metrics, as we have performed in our study, allows for ROI-specific thresholds that act as rough measures of acceptability. Notably, our ROI-specific thresholds are derived from reference standard measurements provided by experts, which were established to have significantly improved segmentation consistency compared with nonexperts.^3^ When stratified by previously defined expert IOV cutoffs, the ROIs with the lowest percentage of observers who were able to cross cutoffs were often tumor volumes. This is consistent with the generally held notion that tumor volumes, which often require domain-specific knowledge, are inherently more difficult to segment than OARs.^21,22^ Echoing the aforementioned results, Bayesian regression analysis demonstrated that the tumor-related ROI type adversely affected segmentation performance. This highlights the need for standardized automated tumor segmentation methods, which so far have been less developed and used than their OAR counterparts.^9^

Interestingly, results were inconsistent and mostly nonsubstantial for the majority of demographic variables across disease sites. Historically, greater institutional support has been perceived to be important for radiotherapy quality.^9^ Therefore, our mostly negative results for proxy variables intuitively linked to greater institutional resource support, such as academic practice and prevalence of radiation oncologist colleagues, are particularly surprising. These findings suggest that the auto-segmentation community should reconsider heuristic choices, such as those based on annotator qualities, when choosing reference segmentations for algorithm development. It may instead be preferable to use consensus segmentations, which have demonstrated quantitatively reliable results as shown in our previous work,^3^ in place of single-annotator segmentations for prospective data collection efforts. Finally, while existing literature regarding observer demographic impact on radiotherapy-related tasks is sparse, it warrants mentioning that one of the few studies in this area found no significant relationship between demographic factors and the resultant quality of radiotherapy plans.^23^ Another study investigating lung disease annotations also demonstrated no impact of observer demographics on segmentation quality.^24^ These studies echo our mostly null results.

While most of the investigated demographic variables were nonsubstantial with large degrees of uncertainties, there were a few results that we believe warrant further discussion. Academic practice in the GYN case was substantially negatively associated with SDSC performance; a nonsubstantial negative association was echoed in most of the disease sites. This could imply, perhaps contrary to common assumptions, that community clinicians produce segmentations more closely aligned with our reference standard and, presumably, more consistent with contouring guidelines. Moreover, White racial self-identification was substantially positively associated with DSC in the H&N case, which exhibited conflicting relationships in other disease sites. It is crucial to emphasize that the association between racial self-identification—a complex social construct which has been drastically simplified in this binary variable—and segmentation performance likely reflects broader institutional or regional conformance to contouring guidelines, rather than a reductive racial skill disparity. Notably, US and European organizations, which would have over-representation of White racial self-identification, have the largest proportion of contouring guideline endorsements.^25^ The heterogeneity within C3RO's categorization of non-US observers might have confounded these relationships. In addition, the presence of a radiation oncologist colleague was substantially positively associated with DSC in the GI case; this positive relationship seemed to hold for most disease sites. These results suggest that clinicians who likely participate in consensus decision making tend to create segmentations closer to our reference standard and thus are likely to adhere to guidelines. Perplexingly, years of practice was found to have a consistently negative (though nonsubstantial) impact on DSC across the various disease sites. This may be because recent clinician graduates are more likely to adhere to contouring guidelines. Finally, our study did not show that treatment of a particular disease site was substantially associated with superior segmentation quality; in fact, it often demonstrated a negative correlation. This seemingly challenges previous findings highlighting the significant role of clinician experience in treatment quality.^4-6^ However, the variable did not assess treatment frequency for the specific site, thereby potentially introducing heterogeneity in its interpretation and ultimately diminishing its utility.

Our study is not without limitations. First, we relied on an existing data set with inherent constraints. While boasting an unprecedented number of radiation oncologist observers, C3RO only principally used a single imaging modality from one representative patient per disease site. While this provides a dedicated reference standard, demographic relationships could change depending on a variety of underlying patient-related factors. Moreover, the C3RO intake survey was self-reported and requested limited demographic information. For example, direct indicators of treatment volume, which have been shown in previous studies to be strongly correlated with patient outcomes,^4^ were not collected because of the high potential for recall bias. Similarly, variables related to the annotator's initial clinical training and current workflow, that is, routine use of contour guidelines/resources/software and access to multiple imaging modalities, would have also likely been highly informative but were not collected. Second, we have relied exclusively on conventional geometry-based metrics of segmentation quality, which have been noted to have flaws in the assessment of radiotherapy-related structures.^1^ Future studies should investigate metrics more closely tied to relevant patient outcomes, such as dose-volume histogram measures. On a related note, how to best define segmentation quality in a quantitative manner, and subsequently how to improve it, remains an open question. We hope to mitigate some of these issues by binarizing our outcome segmentation quality variable and thus calibrating the value relative to a reference standard baseline. We fully acknowledge that this methodology has flaws, principally in that edge cases may be unfairly penalized or rewarded. Furthermore, our definition of a reference standard baseline is a subjective metric derived from our own data set. Specifically, our study presupposes expert consensus segmentations as ideal quality benchmarks. Large deviations from this assumption could indicate that our results simply reflect expected segmentation similarity variations secondary to clinical practice variation. A final limitation of our study lies in our reliance on weakly informative priors for our Bayesian analysis, primarily because of insufficient existing data to extract meaningful priors. Nevertheless, our current data can serve as valuable priors for future Bayesian analyses.

In conclusion, we used an extensive number of radiation oncologist observers in several disease sites to probe trends between common demographic variables and segmentation quality using generalized linear mixed-effects models with Bayesian estimation. Tumor-related structures were, as expected, more difficult to segment than OARs. However, results for demographic factors were mixed and exhibited high uncertainty as evident by large posterior standard deviations and wide HDIs. Surprisingly, there were no obvious recurring relationships for conventionally presumed factors influencing segmentation quality—this may incentivize the research community to reconsider heuristic choices when selecting reference segmentations for auto-segmentation development. While stark variations in quantitative performance among observers compared with our reference standard segmentations can be observed, it is still unclear if and how demographic factors influence segmentation similarity to these benchmarks. Given the anticipated scenario that auto-segmentation algorithms will require humans in the loop in some capacity, these factors are still likely important to understand and should be investigated in prospective analyses of auto-segmentation interaction. Future studies should investigate a greater number of demographic variables (eg, direct indicators of treatment volume), a greater number of patients and imaging modalities, and alternative metrics of segmentation acceptability (eg, dosimetric indicators).
