# Supplementary material for: Associations Between Radiation Oncologist Demographic Factors and Segmentation Similarity Benchmarks: Insights From a Crowd-Sourced Challenge Using Bayesian Estimation
Source: JCO Clin Cancer Inform. Author manuscript; Available in PMC 2024 Jun 30. (PMC11214868; doi:10.1200/CCI.23.00174)
Supplement: Supplementary material [file NIHMS2004281-supplement-Supplementary_material.pdf]

Additional C3RO descriptive information.

Supplementary Table 1. A complete list of the regions of interest (ROIs) used in this study for each disease site with the corresponding number of expert and non-expert segmentations available for each ROI. More information on these structures and the C3RO dataset as a whole can be found in the corresponding data descriptor (https://doi.org/10.1038/s41597-023-02062-w).

Disease site	Type of ROI	ROI	Definition(s)	Number of expert segmentations	Number of non-expert segmentations
Breast	Target volumes	CTV_Ax	Clinical target volume of axillary region	8	115
		CTV_Chestwall	Clinical target volume of chest wall	8	117
		CTV_IMN	Clinical target volume of internal mammary nodes	8	118
		CTV_Sclav_LN	Clinical target volume of supraclavicular lymph nodes	8	119
	OARs	BrachialPlex_L	Brachial plexus left	6	88
		Heart	Heart	7	121
		A_LAD	Left anterior descending artery	7	88
Sarcoma	Target volumes	GTV	Gross tumor volume	5	60
		CTV	Clinical tumor volume	5	48
	OARs	Genitals	Genitalia	4	51
Head and Neck	Target volumes	GTVp	Gross tumor volume primary - right tonsillar fossa	14	59
		GTVn	Gross tumor volume of nodes - nodal spread to level II/III on ipsilateral side (with sternocleidomastoid muscle invaded)	13	60

			and no contralateral nodal involvement		
		CTV1	Clinical target volume (high-risk)	9	45
		CTV2	Clinical target volume (low to intermediate risk)	9	49
	OARs	Brainstem	Brainstem	13	58
		Glnd_Submand_L	Submandibular gland left	13	57
		Glnd_Submand_R	Submandibular gland right	12	52
		Larynx	Larynx	12	57
		Musc_Constrict	All pharyngeal constrictor muscles (superior, middle, and inferior)	11	43
		Parotid_L	Parotid left	13	59
		Parotid_R	Parotid right	13	58
Gynecologic	Target volumes	GTVn	Gross tumor volume of the involved right common iliac lymph node	5	42
		CTVn_4500	Clinical target volume for the elective nodal volumes at risk that will receive 45 Gy	5	40
		CTVp_4500	Clinical target volume primary will receive 45 Gy. This is the combination of “ctv1” and “ctv2” used in many RTOG protocols	5	41
	OARs	Bowel_Small	Small bowel	4	35
Gastrointestinal	Target volumes	CTV_4500	Clinical target volume that will receive 45 Gy	4	25

		CTV_5400	Clinical target volume that will receive 54 Gy	4	23
	OARs	Bag_Bowel	Small and large bowel	4	23

Additional descriptive statistics and exploratory variable analysis.

We calculated descriptive statistics for the radiation oncologist observers used in this study, including median and interquartile range values for numerical variables (total years of practice, number of colleagues) and percentages of binary categorical data (location, self-identified gender, practice type, self-identified race, treat site, academic affiliation, colleague presence). Values for each disease site were calculated separately. Empty entries for numerical values were ignored for these calculations. Descriptive statistics for the non-expert observers used in our study are shown in **Supplementary Table 2**. Descriptive statistics for the expert observers (note: expert segmentations were only used as benchmarks in this study) are shown in **Supplementary Table 3** for completeness. Generally, experts had a larger number of colleagues, US predominance, academic practice type predominance, and colleague presence predominance compared to non-experts. All experts for all disease sites had an academic affiliation and treated the corresponding disease site. Generally, experts had longer total years of practice compared to non-experts, with notable exceptions for the sarcoma and GI cases.

Supplementary Table 2. Descriptive statistics of demographic variables for non-expert observers used in our analysis. Breast, sarcoma, head and neck (H&N), gynecologic (GYN), and gastrointestinal (GI) values were calculated separately. Median (interquartile range) values are shown for numerical variables. Percentages for a given binary class (indicated in parenthesis next to variable) are shown for the categorical variables.

**Variable**	**Breast**	**Sarcoma**	**H&N**	**GYN**	**GI**
Total years of practice	6.00 (3.00,10.00)	7.00 (3.00,11.50)	6.50 (2.25,11.00)	6.50 (4.00,12.50)	8.50 (4.00,15.25)
# of Colleagues	5.00 (2.00,11.00)	4.00 (2.00,10.00)	5.00 (2.00,10.00)	5.00 (2.00,10.25)	4.00 (2.00,10.00)
Location (US)	12.8 %	19.0 %	14.0 %	9.4 %	11.8 %
Gender (F)	39.4 %	42.9 %	56.0 %	37.5 %	52.9 %
Practice type (Academic)	54.3 %	54.8 %	54.0 %	53.1 %	47.1 %
Race white (yes)	43.6 %	42.9 %	46.0 %	34.4 %	47.1 %
Treat site (yes)	91.5 %	69.0 %	92.0 %	84.4 %	82.4 %
Academic affiliation (yes)	47.9 %	54.8 %	64.0 %	65.6 %	64.7 %
Colleague presence (yes)	80.9 %	78.6 %	76.0 %	81.3 %	82.4 %

Supplementary Table 3. Descriptive statistics of demographic variables for expert observers from the C3RO dataset. Breast, sarcoma, head and neck (H&N), gynecologic (GYN), and gastrointestinal (GI) values were calculated separately. Median (interquartile range) values are shown for numerical variables. Percentages for a given binary class (indicated in parenthesis next to variable) are shown for the categorical variables.

Variable	Breast	Sarcoma	H&N	GYN	GI
Total years of practice	7.50 (5.00,13.50)	2.00 (1.00,11.25)	12.00 (5.00,17.50)	9.00 (8.00,10.00)	6.00 (5.50,8.00)
# of Colleagues	15.00 (8.50,35.75)	14.00 (9.75,33.50)	27.00 (13.50,44.00)	7.00 (6.00,10.00)	12.00 (6.50,51.00)
Location (US)	100.0 %	100.0 %	40.0 %	80.0 %	75.0 %
Gender (F)	50.0 %	40.0 %	33.3 %	40.0 %	75.0 %
Practice type (Academic)	100.0 %	80.0 %	93.3 %	100.0 %	75.0 %
Race white (yes)	62.5 %	40.0 %	46.7 %	80.0 %	100.0 %
Treat site (yes)	100.0 %	100.0 %	100.0 %	100.0 %	100.0 %
Academic affiliation (yes)	100.0 %	100.0 %	100.0 %	100.0 %	100.0 %
Colleague presence (yes)	87.5 %	100.0 %	93.3 %	100.0 %	100.0 %

Using the demographic variables, we then performed an exploratory analysis to determine if any variables exhibited high correlations within the non-expert observers. A Spearman's rank correlation was employed since it could utilize continuous numerical values and binary data simultaneously. Correlation heatmaps for each disease site are shown for each disease site in **Supplementary Figures 1-5**. After the exploratory analysis, academic affiliation was chosen to be excluded from the regression analysis due to its high correlation with practice type to enable greater model parsimony.

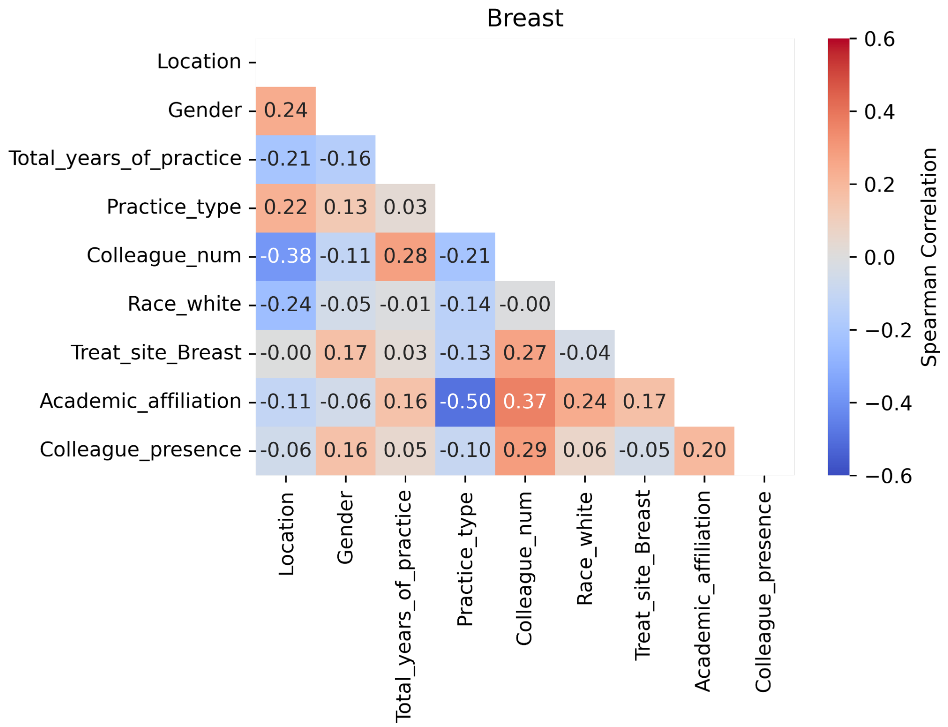

Supplementary Figure 1. Correlation heatmap for the breast case.

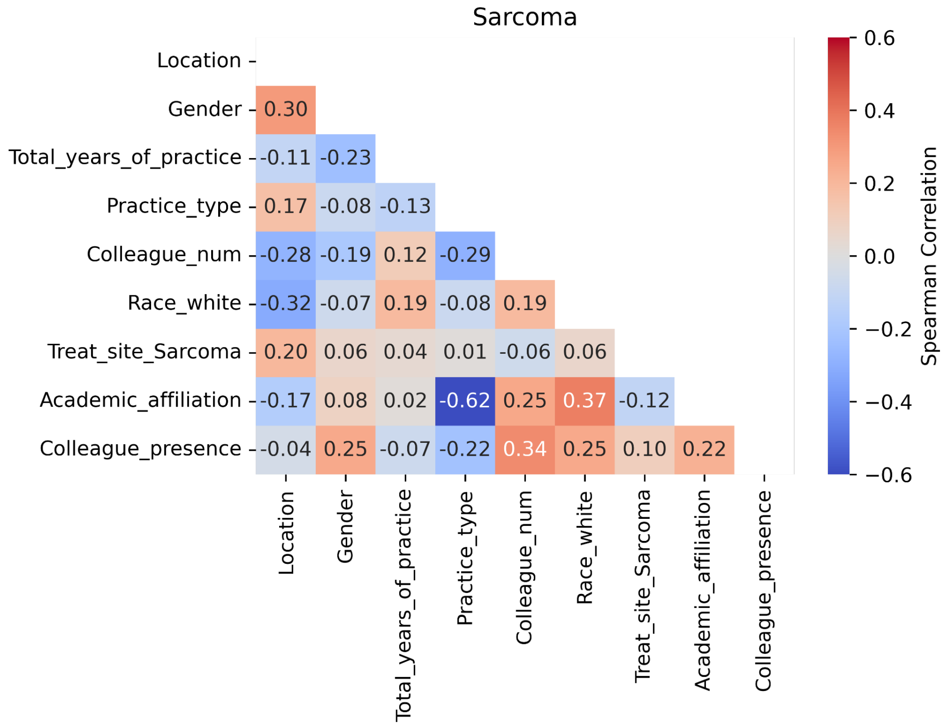

Supplementary Figure 2. Correlation heatmap for the sarcoma case.

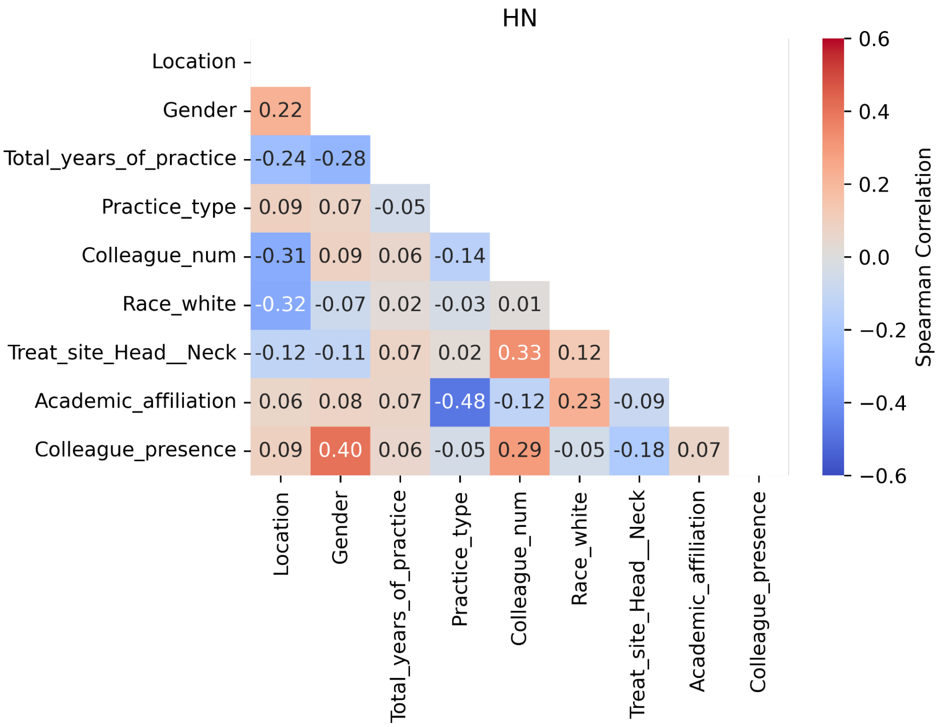

Supplementary Figure 3. Correlation heatmap for the head and neck (HN) case.

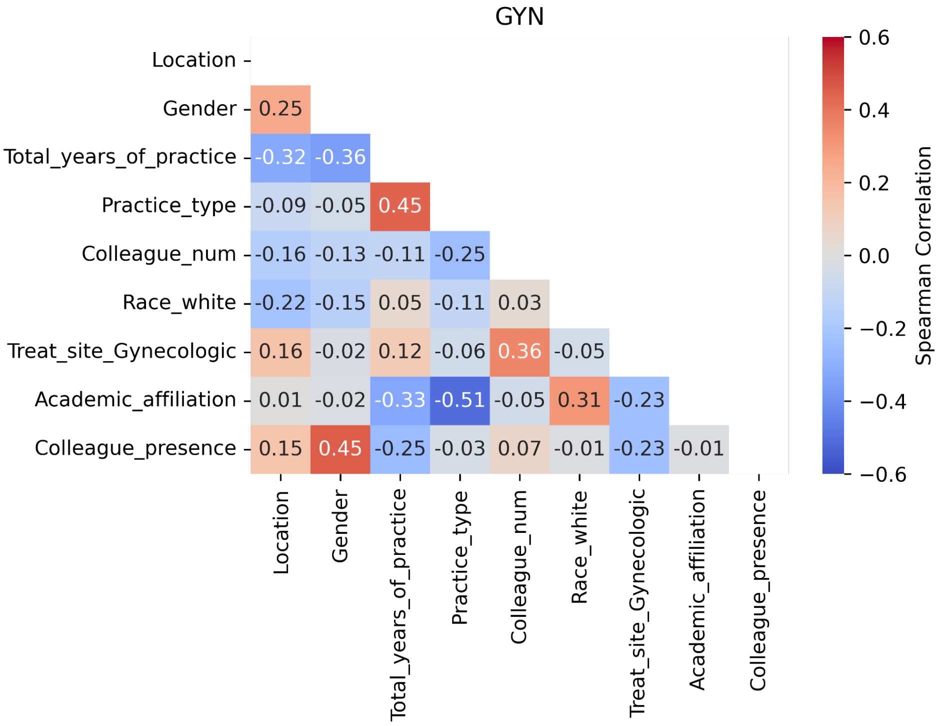

Supplementary Figure 4. Correlation heatmap for the gynecologic (GYN) case.

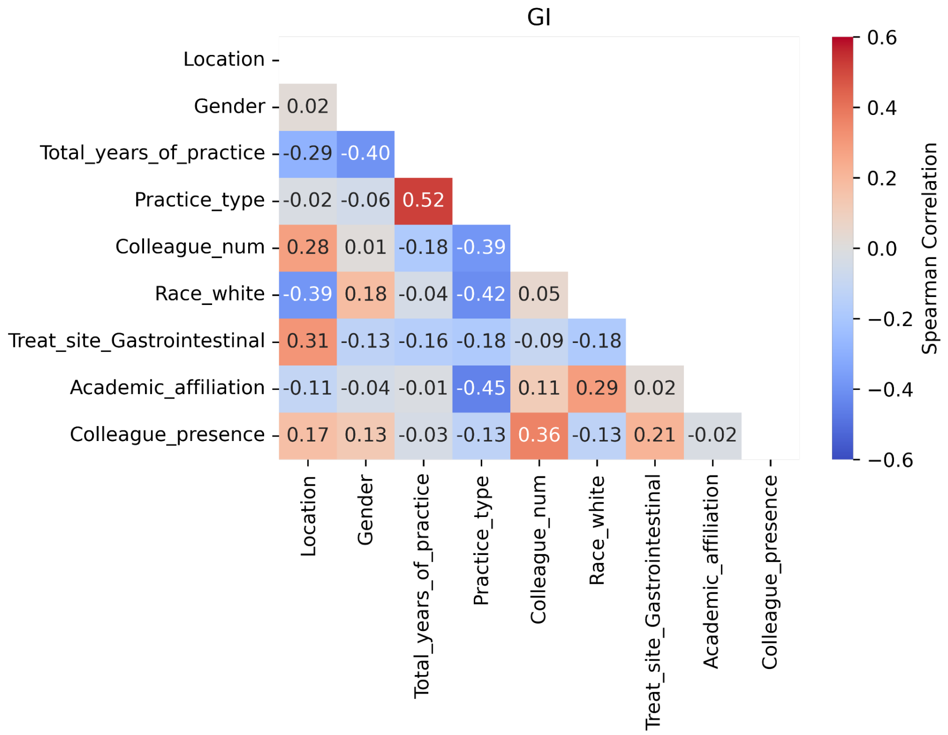

Supplementary Figure 5. Correlation heatmap for the gastrointestinal (GI) case.

Additional plots.

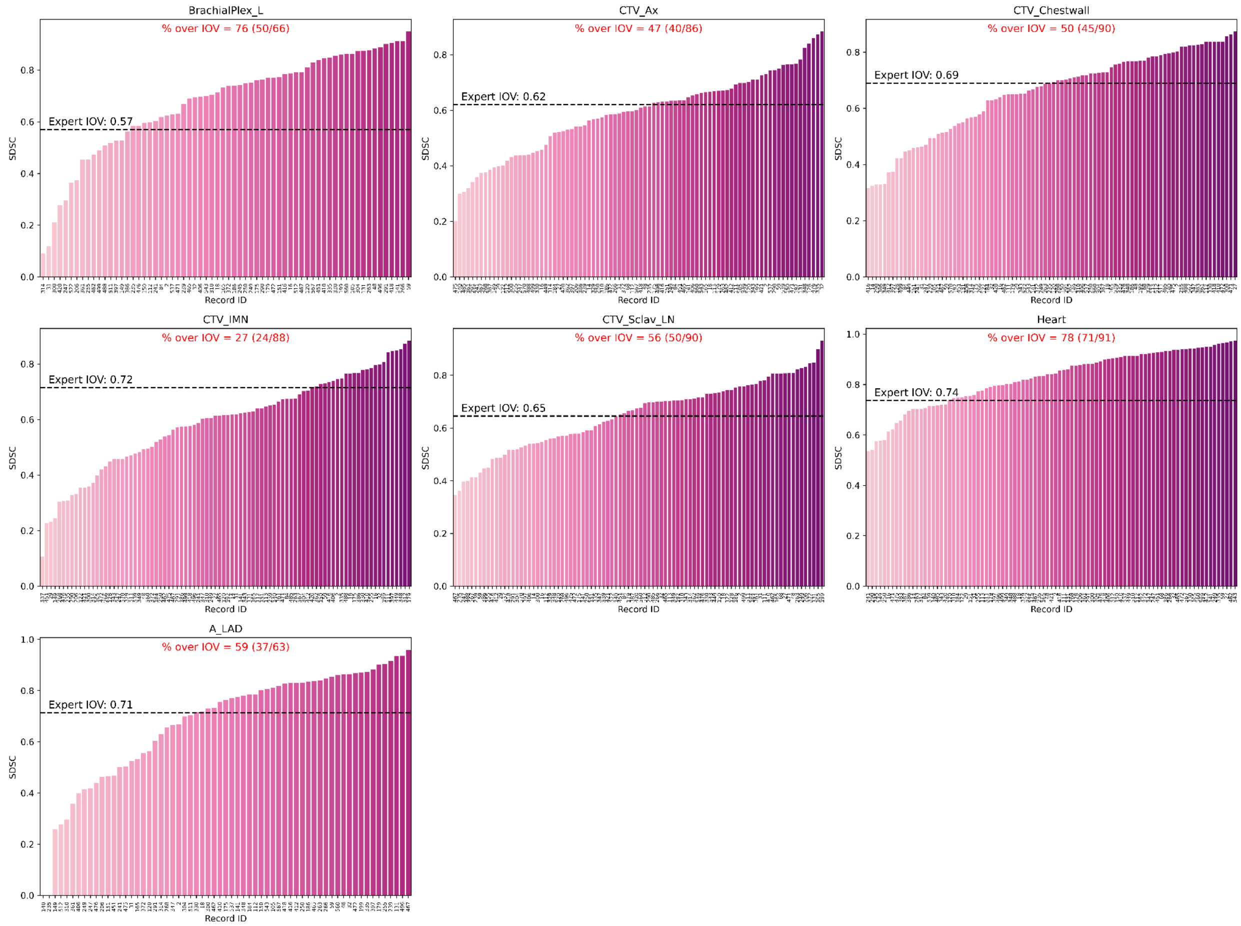

Supplementary Figure 6. Barplots of individual observer segmentation performance vs. reference standard for the breast case using surface Dice similarity coefficient (SDSC). The reference standard segmentation is the consensus segmentation of all experts as derived from Simultaneous Truth and Performance Level Estimation (STAPLE). Black dotted lines indicate median expert interobserver SDSC for a corresponding region of interest. The percentage of observers that were able to cross the expert interobserver variability (IOV) cutoff are also shown in red above each plot.

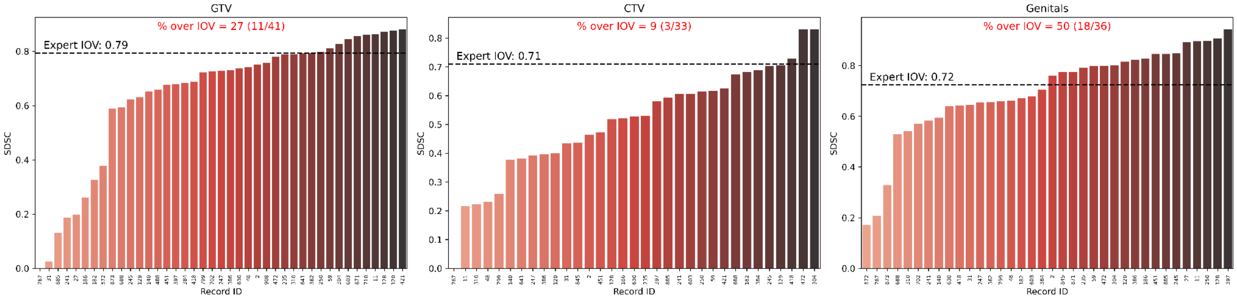

Supplementary Figure 7. Barplots of individual observer segmentation performance vs. reference standard for sarcoma case using surface Dice similarity coefficient (SDSC). The reference standard segmentation is the consensus segmentation of all experts as derived from Simultaneous Truth and Performance Level Estimation (STAPLE). Black dotted lines indicate median expert interobserver SDSC for a corresponding region of interest. The percentage of observers that were able to cross the expert interobserver variability (IOV) cutoff are also shown in red above each plot.

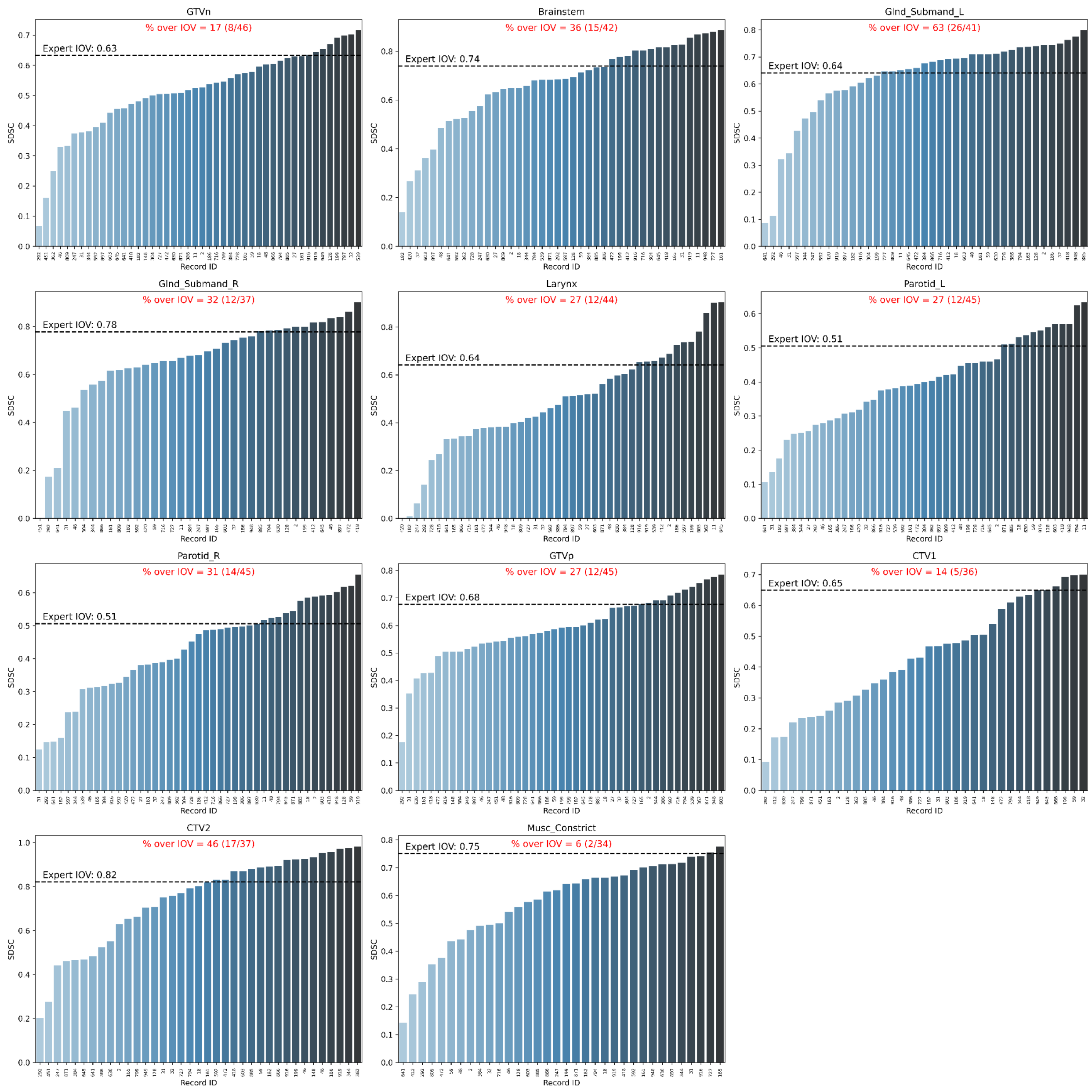

Supplementary Figure 8. Barplots of individual observer segmentation performance vs. reference standard for head and neck case using surface Dice similarity coefficient (SDSC). The reference standard segmentation is the consensus segmentation of all experts as derived from Simultaneous Truth and Performance Level Estimation (STAPLE). Black dotted lines indicate median expert interobserver SDSC for a corresponding region of interest. The percentage of observers that were able to cross the expert interobserver variability (IOV) cutoff are also shown in red above each plot.

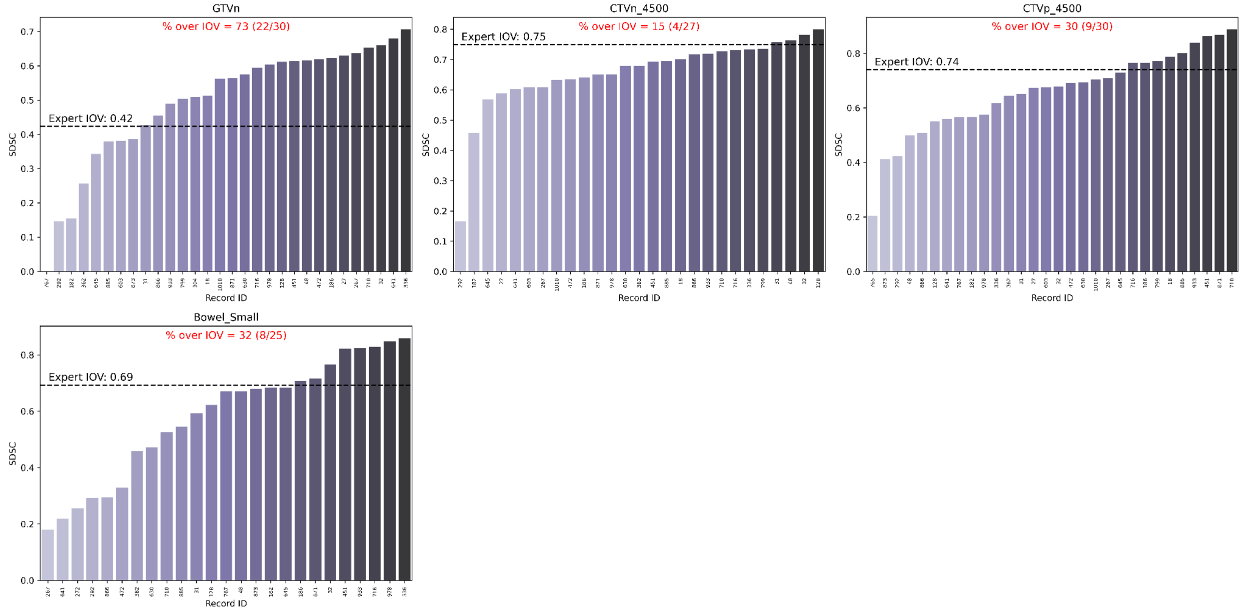

Supplementary Figure 9. Barplots of individual observer segmentation performance vs. reference standard for gynecologic case using surface Dice similarity coefficient (SDSC). The reference standard segmentation is the consensus segmentation of all experts as derived from Simultaneous Truth and Performance Level Estimation (STAPLE). Black dotted lines indicate median expert interobserver SDSC for a corresponding region of interest. The percentage of observers that were able to cross the expert interobserver variability (IOV) cutoff are also shown in red above each plot.

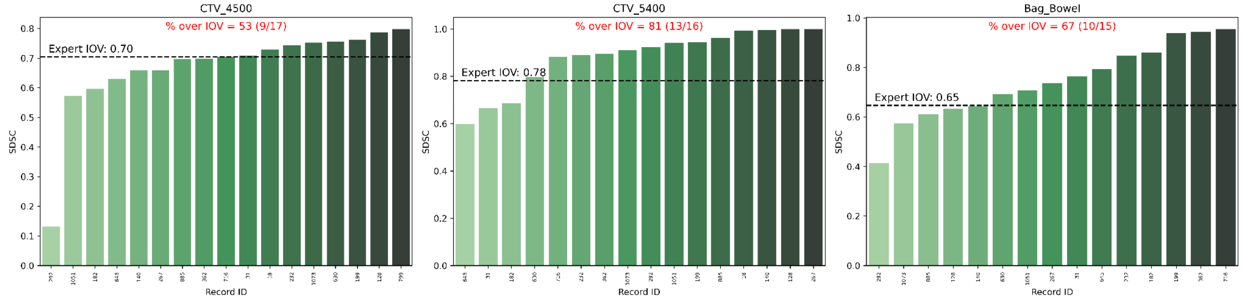

Supplementary Figure 10. Barplots of individual observer segmentation performance vs. reference standard for gastrointestinal case using surface Dice similarity coefficient (SDSC). The reference standard segmentation is the consensus segmentation of all experts as derived from Simultaneous Truth and Performance Level Estimation (STAPLE). Black dotted lines indicate median expert interobserver SDSC for a corresponding region of interest. The percentage of observers that were able to cross the expert interobserver variability (IOV) cutoff are also shown in red above each plot.

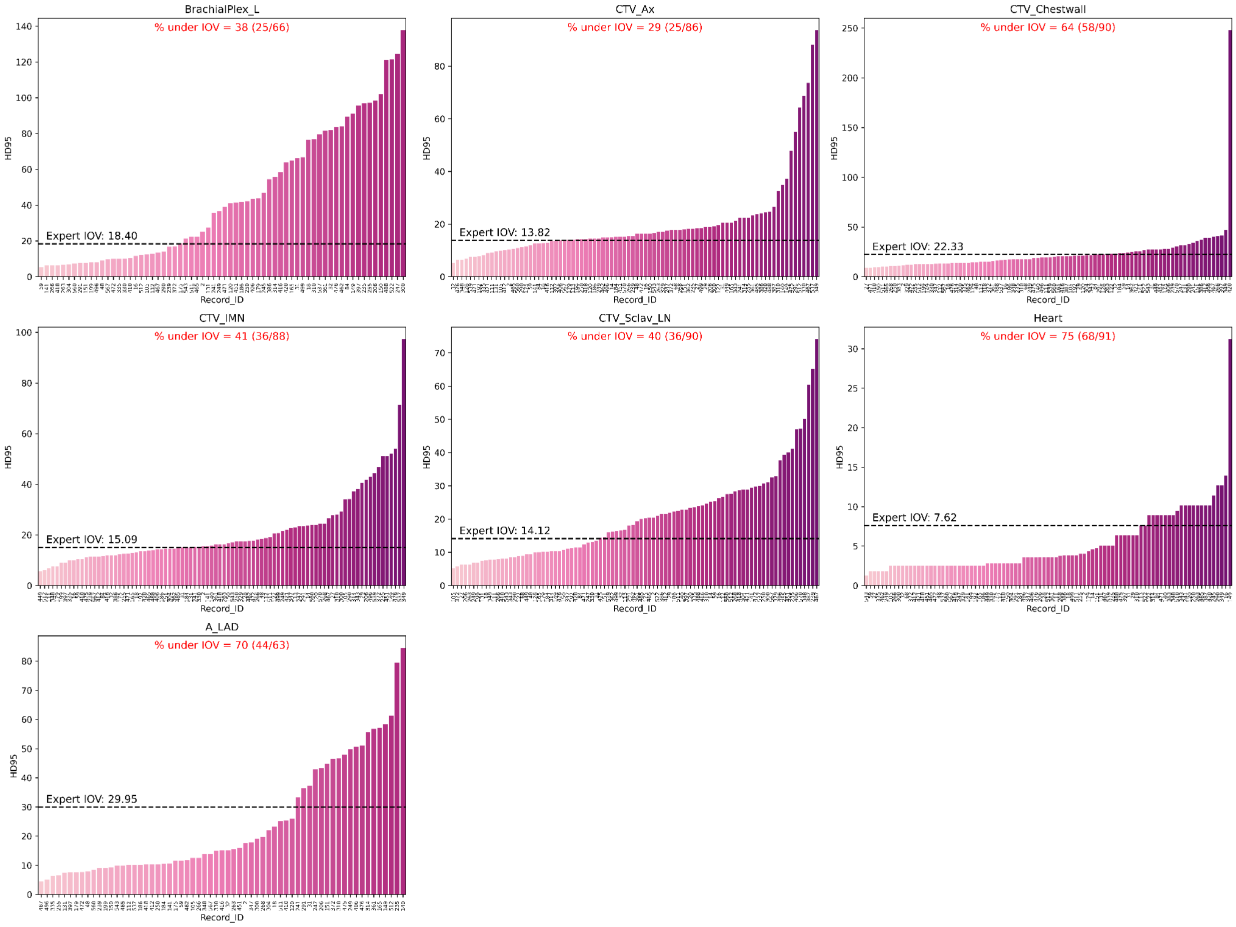

Supplementary Figure 11. Barplots of individual observer segmentation performance vs. reference standard for the breast case using 95% Hausdorff distance (HD95). The reference standard segmentation is the consensus segmentation of all experts as derived from Simultaneous Truth and Performance Level Estimation (STAPLE). Black dotted lines indicate median expert interobserver HD95 for a corresponding region of interest. The percentage of observers that were able to cross the expert interobserver variability (IOV) cutoff are also shown in red above each plot.

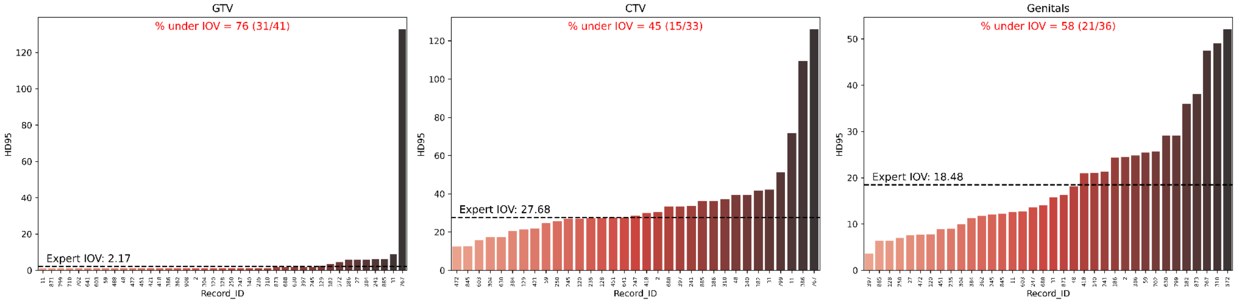

Supplementary Figure 12. Barplots of individual observer segmentation performance vs. reference standard for the sarcoma case using 95% Hausdorff distance (HD95). The reference standard segmentation is the consensus segmentation of all experts as derived from Simultaneous Truth and Performance Level Estimation (STAPLE). Black dotted lines indicate median expert interobserver HD95 for a corresponding region of interest. The percentage of observers that were able to cross the expert interobserver variability (IOV) cutoff are also shown in red above each plot.

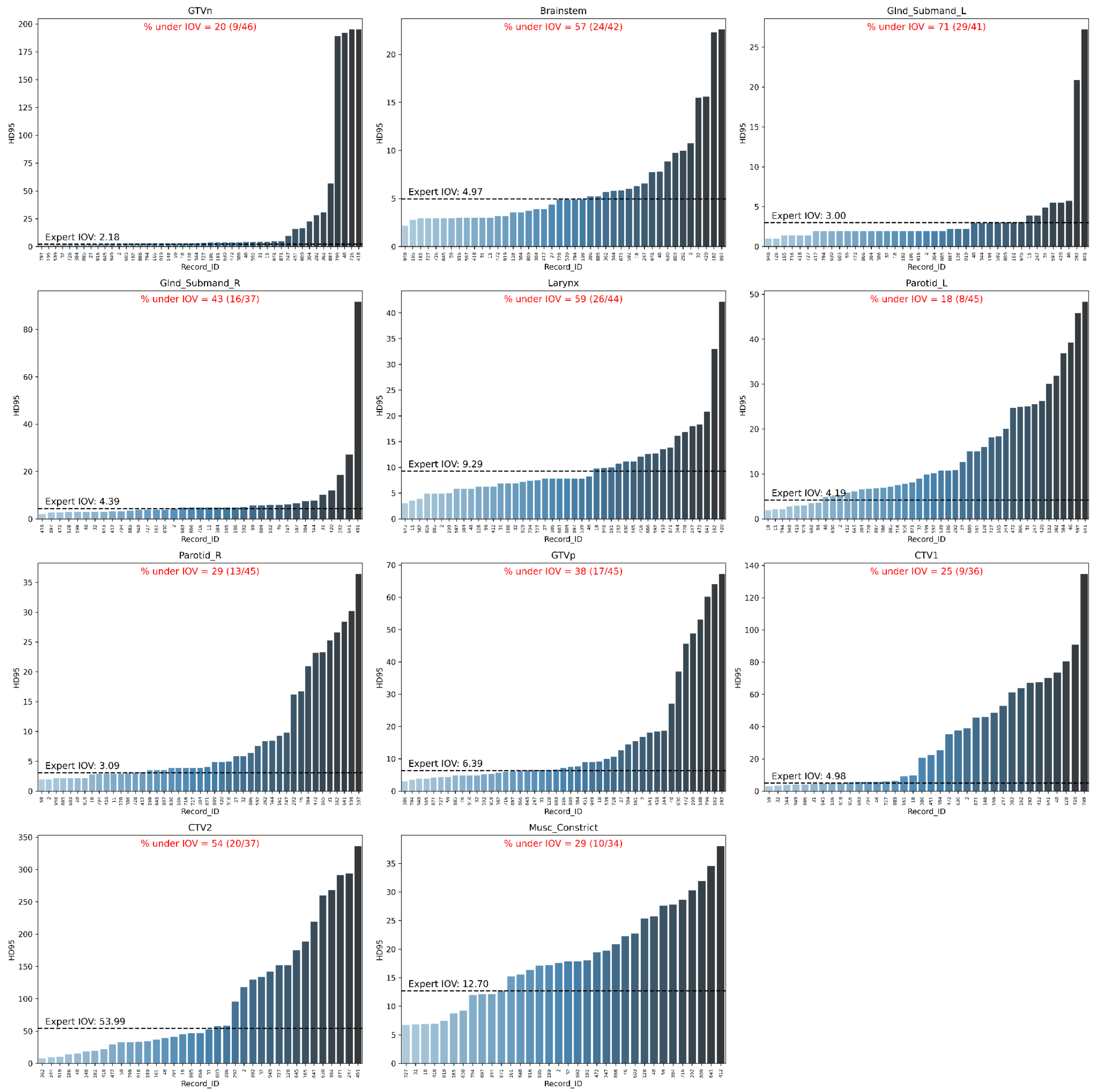

Supplementary Figure 13. Barplots of individual observer segmentation performance vs. reference standard for the head and neck case using 95% Hausdorff distance (HD95). The reference standard segmentation is the consensus segmentation of all experts as derived from Simultaneous Truth and Performance Level Estimation (STAPLE). Black dotted lines indicate median expert interobserver HD95 for a corresponding region of interest. The percentage of observers that were able to cross the expert interobserver variability (IOV) cutoff are also shown in red above each plot.

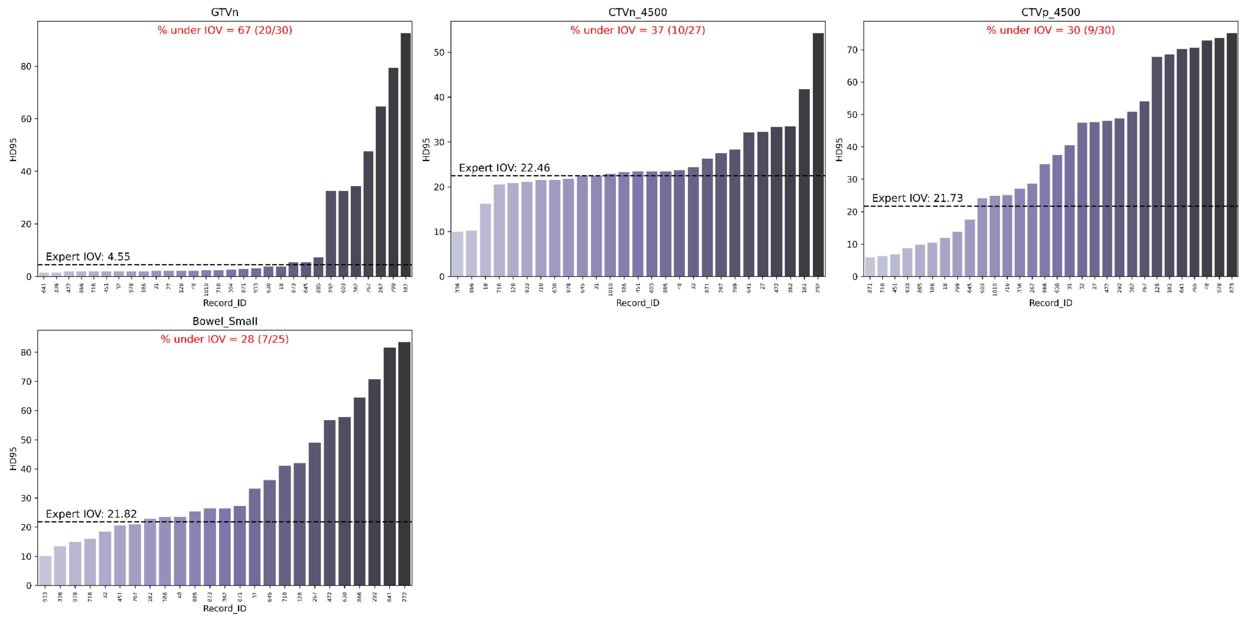

Supplementary Figure 14. Barplots of individual observer segmentation performance vs. reference standard for the gynecologic case using 95% Hausdorff distance (HD95). The reference standard segmentation is the consensus segmentation of all experts as derived from Simultaneous Truth and Performance Level Estimation (STAPLE). Black dotted lines indicate median expert interobserver HD95 for a corresponding region of interest. The percentage of observers that were able to cross the expert interobserver variability (IOV) cutoff are also shown in red above each plot.

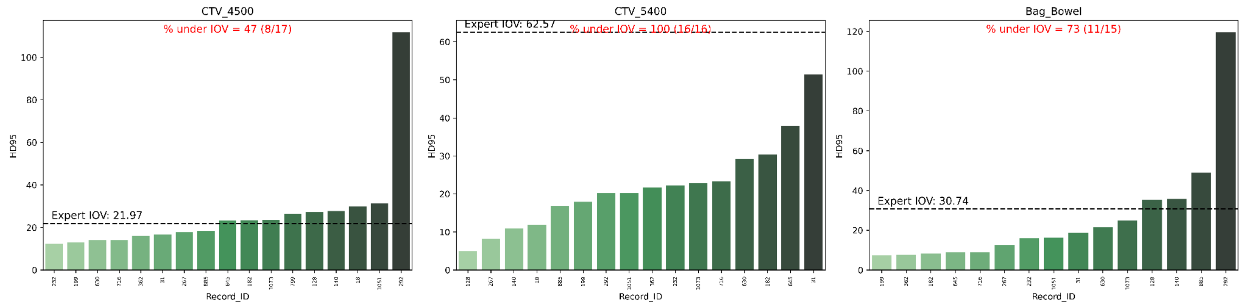

Supplementary Figure 15. Barplots of individual observer segmentation performance vs. reference standard for the gastrointestinal case using 95% Hausdorff distance (HD95). The reference standard segmentation is the consensus segmentation of all experts as derived from Simultaneous Truth and Performance Level Estimation (STAPLE). Black dotted lines indicate median expert interobserver HD95 for a corresponding region of interest. The percentage of observers that were able to cross the expert interobserver variability (IOV) cutoff are also shown in red above each plot.

Additional information on Bayesian regression.

Results for the regression models using the 95% Hausdorff distance (HD95) as the dependent variable are shown in **Supplementary Table 4**. Notably, as opposed to the other metrics (DSC and SDSC), smaller HD95 values are better, so the target output was coded as 0 if the observation was above the IOV cutoff and 1 if the observation was below the IOV cutoff.

Markov chain Monte Carlo convergence metric summary values were calculated for each model. **Supplementary Tables 5-19** display the Monte Carlo Standard Error of the mean (msce_mean), Monte Carlo Standard Error of the standard deviation (msce_sd), Effective Sample Size for the bulk of the distribution (ess_bulk), Effective Sample Size for the tail of the distribution (ess_tail), and the Gelman-Rubin statistic (r_hat) for each variable.

Supplementary Table 4. Generalized linear mixed-effects models with Bayesian estimation results using binarized Hausdorff distance as the outcome variable. Model coefficient values are shown for each variable. Reference variable for categorical variables are shown in brackets next to variable name. Sign value in posterior mean indicates positive or negative correlation of variable with outcome. Posterior standard deviation (SD) indicates uncertainty around posterior mean. 89% highest density interval (HDI) is shown in parenthesis after posterior mean. * Bolded variables indicate HDI does not contain zero and is considered to have a substantial impact on the outcome measure of interest.

	**Breast**		**Sarcoma**		**Head and Neck**		**Gynecologic**		**Gastrointestinal**	
**Variables**	Mean (HDI)	SD	Mean (HDI)	SD	Mean (HDI)	SD	Mean (HDI)	SD	Mean (HDI)	SD
Intercept	0.19 (-0.53,0.91)	0.45	2.25 (-0.53,4.98)	1.77	-0.39 (-1.58,0.78)	0.74	-0.51 (-3.31,2.35)	1.80	-1.69 (-6.79,3.49)	3.36
ROI type [Tumor]	**-0.79 (-1.08,-0.50)***	0.18	0.27 (-0.60,1.15)	0.55	**-0.49 (-0.84,-0.12)***	0.23	**1.12 (0.13,2.11)***	0.62	0.16 (-1.32,1.71)	0.95
Location [US]	-0.37 (-0.89,0.16)	0.33	0.38 (-1.69,2.46)	1.32	0.09 (-0.76,0.94)	0.54	-0.90 (-3.26,1.53)	1.55	-1.54 (-5.83,2.79)	2.74
Gender [Female]	-0.23 (-0.59,0.12)	0.22	-0.88 (-2.41,0.74)	1.01	-0.24 (-0.89,0.39)	0.40	-1.06 (-2.67,0.56)	1.03	**3.49 (0.95,5.94)***	1.62
Years of practice	0.01 (-0.16,0.17)	0.10	**1.16 (0.25,2.08)***	0.60	-0.11 (-0.38,0.19)	0.18	-0.40 (-1.19,0.40)	0.50	0.20 (-1.57,1.84)	1.08
Practice type [academic]	0.07 (-0.26,0.40)	0.21	-1.21 (-2.65,0.25)	0.93	0.30 (-0.23,0.84)	0.34	-0.66 (-2.11,0.85)	0.96	-2.65 (-6.38,1.09)	2.37
# of Colleagues	0.00 (-0.17,0.17)	0.11	0.05 (-0.72,0.87)	0.50	0.07 (-0.25,0.39)	0.20	0.06 (-0.65,0.73)	0.44	**2.73 (0.51,4.89)***	1.39
Colleague presence [yes]	0.08 (-0.36,0.50)	0.27	-0.67 (-2.62,1.24)	1.23	0.10 (-0.62,0.82)	0.45	-0.22 (-2.25,1.89)	1.32	1.74 (-1.09,4.53)	1.79
Race [white]	-0.07 (-0.40,0.25)	0.21	0.33 (-1.13,1.85)	0.95	0.51 (-0.06,1.07)	0.36	0.26 (-1.15,1.69)	0.91	-0.90 (-3.56,1.71)	1.68
Treat disease site [yes]	0.43 (-0.18,1.02)	0.38	-0.23 (-1.75,1.33)	0.98	-0.30 (-1.33,0.73)	0.65	-0.09 (-2.14,1.80)	1.26	3.01 (-1.08,6.81)	2.55
Random effect variance	0.36 (0.00,0.60)	0.19	1.99 (0.75,3.18)	0.79	0.83 (0.50,1.15)	0.21	1.69 (0.61,2.75)	0.70	1.40 (0.00,2.89)	1.18

Supplementary Table 5. Convergence parameters for the breast case using binarized DSC as the dependent variable.

Case	Metric	Variable	mcse_mean	mcse_sd	ess_bulk	ess_tail	r_hat
Breast	DSC_binary	Intercept	0.003	0.002	29568	22936	1
Breast	DSC_binary	C(ROI_type)[Tumor]	0.001	0.001	39951	24478	1
Breast	DSC_binary	C(Location)[US]	0.002	0.002	24293	20372	1
Breast	DSC_binary	C(Gender, Treatment("Male"))[Female]	0.002	0.001	25101	19633	1
Breast	DSC_binary	Total_years_of_practice	0.001	0.001	24629	19463	1
Breast	DSC_binary	C(Practice_type, Treatment("Non-academic"))[Academic]	0.001	0.001	25523	18624	1
Breast	DSC_binary	Colleague_num	0.001	0.001	27153	23344	1
Breast	DSC_binary	C(Colleague_presence, Treatment("No"))[Yes]	0.002	0.002	26875	21606	1
Breast	DSC_binary	C(Race_white, Treatment("Unchecked"))[Checked]	0.001	0.001	25907	18128	1
Breast	DSC_binary	C(Treat_site_Breast, Treatment("Unchecked"))[Checked]	0.003	0.002	26558	21617	1
Breast	DSC_binary	1|Record_ID_sigma	0.002	0.002	8348	10181	1

Supplementary Table 6. Convergence parameters for the breast case using binarized SDSC as the dependent variable.

Case	Metric	Variable	mcse_mean	mcse_sd	ess_bulk	ess_tail	r_hat
Breast	SDSC_binary	Intercept	0.002	0.002	55399	31595	1
Breast	SDSC_binary	C(ROI_type)[Tumor]	0.001	0.001	63876	28492	1
Breast	SDSC_binary	C(Location)[US]	0.002	0.002	50284	31121	1
Breast	SDSC_binary	C(Gender, Treatment("Male"))[Female]	0.001	0.001	50534	31443	1
Breast	SDSC_binary	Total_years_of_practice	0	0.001	54594	32974	1
Breast	SDSC_binary	C(Practice_type, Treatment("Non-academic"))[Academic]	0.001	0.001	50801	31170	1
Breast	SDSC_binary	Colleague_num	0.001	0.001	50473	30704	1
Breast	SDSC_binary	C(Colleague_presence, Treatment("No"))[Yes]	0.001	0.001	51137	30348	1
Breast	SDSC_binary	C(Race_white, Treatment("Unchecked"))[Checked]	0.001	0.001	51749	31319	1
Breast	SDSC_binary	C(Treat_site_Breast, Treatment("Unchecked"))[Checked]	0.002	0.002	50551	30016	1
Breast	SDSC_binary	1|Record_ID_sigma	0.002	0.001	9414	10873	1

Supplementary Table 7. Convergence parameters for the breast case using binarized HD95 as the dependent variable.

Case	Metric	Variable	mcse_mean	mcse_sd	ess_bulk	ess_tail	r_hat
Breast	HD95_binary	Intercept	0.003	0.003	24152	18729	1
Breast	HD95_binary	C(ROI_type)[Tumor]	0.001	0.001	35793	22164	1
Breast	HD95_binary	C(Location)[US]	0.002	0.002	22618	18661	1
Breast	HD95_binary	C(Gender, Treatment("Male"))[Female]	0.002	0.001	20332	15206	1
Breast	HD95_binary	Total_years_of_practice	0.001	0.001	22036	17778	1
Breast	HD95_binary	C(Practice_type, Treatment("Non-academic"))[Academic]	0.001	0.001	22238	17476	1
Breast	HD95_binary	Colleague_num	0.001	0.001	22862	16972	1
Breast	HD95_binary	C(Colleague_presence, Treatment("No"))[Yes]	0.002	0.001	21894	18182	1
Breast	HD95_binary	C(Race_white, Treatment("Unchecked"))[Checked]	0.001	0.002	22159	16608	1
Breast	HD95_binary	C(Treat_site_Breast, Treatment("Unchecked"))[Checked]	0.002	0.002	23740	19189	1
Breast	HD95_binary	1|Record_ID_sigma	0.002	0.002	6539	11367	1

Supplementary Table 8. Convergence parameters for the sarcoma case using binarized DSC as the dependent variable.

Case	Metric	Variable	mcse_mean	mcse_sd	ess_bulk	ess_tail	r_hat
Sarcoma	DSC_binary	Intercept	0.013	0.012	12890	8384	1
Sarcoma	DSC_binary	C(ROI_type)[Tumor]	0.004	0.003	21729	17369	1
Sarcoma	DSC_binary	C(Location)[US]	0.009	0.007	14397	7301	1
Sarcoma	DSC_binary	C(Gender, Treatment("Male"))[Female]	0.006	0.006	16230	12533	1
Sarcoma	DSC_binary	Total_years_of_practice	0.008	0.01	5516	2137	1
Sarcoma	DSC_binary	C(Practice_type, Treatment("Non-academic"))[Academic]	0.013	0.018	5460	2422	1
Sarcoma	DSC_binary	Colleague_num	0.006	0.005	5777	2949	1
Sarcoma	DSC_binary	C(Colleague_presence, Treatment("No"))[Yes]	0.012	0.011	8407	4670	1
Sarcoma	DSC_binary	C(Race_white, Treatment("Unchecked"))[Checked]	0.011	0.009	6580	2933	1
Sarcoma	DSC_binary	C(Treat_site_Sarcoma, Treatment("Unchecked"))[Checked]	0.017	0.018	4992	2352	1
Sarcoma	DSC_binary	1|Record_ID_sigma	0.015	0.014	3533	1683	1

Supplementary Table 9. Convergence parameters for the sarcoma case using binarized SDSC as the dependent variable.

Case	Metric	Variable	mcse_mean	mcse_sd	ess_bulk	ess_tail	r_hat
Sarcoma	SDSC_binary	Intercept	0.018	0.015	16091	15281	1
Sarcoma	SDSC_binary	C(ROI_type)[Tumor]	0.006	0.004	17377	21681	1
Sarcoma	SDSC_binary	C(Location)[US]	0.014	0.011	14959	16472	1
Sarcoma	SDSC_binary	C(Gender, Treatment("Male"))[Female]	0.01	0.008	17795	17283	1
Sarcoma	SDSC_binary	Total_years_of_practice	0.004	0.003	19510	20071	1
Sarcoma	SDSC_binary	C(Practice_type, Treatment("Non-academic"))[Academic]	0.011	0.008	16177	17390	1
Sarcoma	SDSC_binary	Colleague_num	0.006	0.005	16206	15753	1
Sarcoma	SDSC_binary	C(Colleague_presence, Treatment("No"))[Yes]	0.014	0.011	14643	12760	1
Sarcoma	SDSC_binary	C(Race_white, Treatment("Unchecked"))[Checked]	0.01	0.009	17995	17831	1
Sarcoma	SDSC_binary	C(Treat_site_Sarcoma, Treatment("Unchecked"))[Checked]	0.01	0.009	17504	14253	1
Sarcoma	SDSC_binary	1|Record_ID_sigma	0.014	0.01	6153	10127	1

Supplementary Table 10. Convergence parameters for the sarcoma case using binarized HD95 as the dependent variable.

Case	Metric	Variable	mcse_mean	mcse_sd	ess_bulk	ess_tail	r_hat
Sarcoma	HD95_binary	Intercept	0.011	0.009	27999	22010	1
Sarcoma	HD95_binary	C(ROI_type)[Tumor]	0.002	0.003	75926	29778	1
Sarcoma	HD95_binary	C(Location)[US]	0.008	0.007	29436	23671	1
Sarcoma	HD95_binary	C(Gender, Treatment("Male"))[Female]	0.006	0.005	27873	21934	1
Sarcoma	HD95_binary	Total_years_of_practice	0.004	0.003	28209	20462	1
Sarcoma	HD95_binary	C(Practice_type, Treatment("Non-academic"))[Academic]	0.006	0.005	28291	22194	1
Sarcoma	HD95_binary	Colleague_num	0.003	0.003	30533	24493	1
Sarcoma	HD95_binary	C(Colleague_presence, Treatment("No"))[Yes]	0.007	0.006	28484	23525	1
Sarcoma	HD95_binary	C(Race_white, Treatment("Unchecked"))[Checked]	0.006	0.005	30239	24810	1
Sarcoma	HD95_binary	C(Treat_site_Sarcoma, Treatment("Unchecked"))[Checked]	0.006	0.005	30583	22840	1
Sarcoma	HD95_binary	1|Record_ID_sigma	0.009	0.006	8326	12170	1

Supplementary Table 11. Convergence parameters for the head and neck case using binarized DSC as the dependent variable.

Case	Metric	Variable	mcse_mean	mcse_sd	ess_bulk	ess_tail	r_hat
HN	DSC_binary	Intercept	0.004	0.004	37705	30054	1
HN	DSC_binary	C(ROI_type)[Tumor]	0.001	0.001	65671	30350	1
HN	DSC_binary	C(Location)[US]	0.004	0.003	31102	25877	1
HN	DSC_binary	C(Gender, Treatment("Male"))[Female]	0.003	0.002	33725	26508	1
HN	DSC_binary	Total_years_of_practice	0.001	0.001	34766	28060	1
HN	DSC_binary	C(Practice_type, Treatment("Non-academic"))[Academic]	0.002	0.002	35287	29790	1
HN	DSC_binary	Colleague_num	0.001	0.001	31819	26266	1
HN	DSC_binary	C(Colleague_presence, Treatment("No"))[Yes]	0.003	0.002	33815	28420	1
HN	DSC_binary	C(Race_white, Treatment("Unchecked"))[Checked]	0.002	0.002	32625	27214	1
HN	DSC_binary	C(Treat_site_Head__Neck, Treatment("Unchecked"))[Checked]	0.004	0.004	33875	26945	1
HN	DSC_binary	1|Record_ID_sigma	0.002	0.001	13010	19892	1

Supplementary Table 12. Convergence parameters for the head and neck case using binarized SDSC as the dependent variable.

Case	Metric	Variable	mcse_mean	mcse_sd	ess_bulk	ess_tail	r_hat
HN	SDSC_binary	Intercept	0.005	0.004	28560	24200	1
HN	SDSC_binary	C(ROI_type)[Tumor]	0.001	0.001	50955	27532	1
HN	SDSC_binary	C(Location)[US]	0.004	0.003	23345	19662	1
HN	SDSC_binary	C(Gender, Treatment("Male"))[Female]	0.003	0.002	23809	20550	1
HN	SDSC_binary	Total_years_of_practice	0.001	0.001	25764	21664	1
HN	SDSC_binary	C(Practice_type, Treatment("Non-academic"))[Academic]	0.002	0.002	24808	21228	1
HN	SDSC_binary	Colleague_num	0.001	0.001	23480	21336	1
HN	SDSC_binary	C(Colleague_presence, Treatment("No"))[Yes]	0.003	0.003	22892	19482	1
HN	SDSC_binary	C(Race_white, Treatment("Unchecked"))[Checked]	0.002	0.002	25718	22428	1
HN	SDSC_binary	C(Treat_site_Head__Neck, Treatment("Unchecked"))[Checked]	0.004	0.004	26493	21941	1
HN	SDSC_binary	1|Record_ID_sigma	0.002	0.002	9903	15345	1

Supplementary Table 13. Convergence parameters for the head and neck case using binarized HD95 as the dependent variable.

Case	Metric	Variable	mcse_mean	mcse_sd	ess_bulk	ess_tail	r_hat
HN	HD95_binary	Intercept	0.004	0.003	37774	27835	1
HN	HD95_binary	C(ROI_type)[Tumor]	0.001	0.001	65404	28231	1
HN	HD95_binary	C(Location)[US]	0.003	0.003	33464	26118	1
HN	HD95_binary	C(Gender, Treatment("Male"))[Female]	0.002	0.002	33854	27950	1
HN	HD95_binary	Total_years_of_practice	0.001	0.001	33860	26487	1
HN	HD95_binary	C(Practice_type, Treatment("Non-academic"))[Academic]	0.002	0.002	34652	27062	1
HN	HD95_binary	Colleague_num	0.001	0.001	34162	27109	1
HN	HD95_binary	C(Colleague_presence, Treatment("No"))[Yes]	0.002	0.002	33495	27894	1
HN	HD95_binary	C(Race_white, Treatment("Unchecked"))[Checked]	0.002	0.001	35036	28362	1
HN	HD95_binary	C(Treat_site_Head__Neck, Treatment("Unchecked"))[Checked]	0.003	0.003	34832	26548	1
HN	HD95_binary	1|Record_ID_sigma	0.002	0.001	13121	20074	1

Supplementary Table 14. Convergence parameters for the gynecologic case using binarized DSC as the dependent variable.

Case	Metric	Variable	mcse_mean	mcse_sd	ess_bulk	ess_tail	r_hat
GYN	DSC_binary	Intercept	0.009	0.008	19783	16063	1
GYN	DSC_binary	C(ROI_type)[Tumor]	0.003	0.003	39118	24318	1
GYN	DSC_binary	C(Location)[US]	0.008	0.007	14987	12676	1
GYN	DSC_binary	C(Gender, Treatment("Male"))[Female]	0.005	0.005	15205	13749	1
GYN	DSC_binary	Total_years_of_practice	0.003	0.004	13143	8731	1
GYN	DSC_binary	C(Practice_type, Treatment("Non-academic"))[Academic]	0.005	0.006	15083	11035	1
GYN	DSC_binary	Colleague_num	0.002	0.002	17355	17195	1
GYN	DSC_binary	C(Colleague_presence, Treatment("No"))[Yes]	0.007	0.006	15862	12871	1
GYN	DSC_binary	C(Race_white, Treatment("Unchecked"))[Checked]	0.004	0.003	21056	17800	1
GYN	DSC_binary	C(Treat_site_Gynecologic, Treatment("Unchecked"))[Checked]	0.007	0.006	16107	13609	1
GYN	DSC_binary	1|Record_ID_sigma	0.006	0.004	6940	8262	1

Supplementary Table 15. Convergence parameters for the gynecologic case using binarized SDSC as the dependent variable.

Case	Metric	Variable	mcse_mean	mcse_sd	ess_bulk	ess_tail	r_hat
GYN	SDSC_binary	Intercept	0.012	0.01	18014	12241	1
GYN	SDSC_binary	C(ROI_type)[Tumor]	0.003	0.004	31914	19393	1
GYN	SDSC_binary	C(Location)[US]	0.01	0.008	16197	15236	1
GYN	SDSC_binary	C(Gender, Treatment("Male"))[Female]	0.007	0.007	14827	12270	1
GYN	SDSC_binary	Total_years_of_practice	0.004	0.003	13708	9130	1
GYN	SDSC_binary	C(Practice_type, Treatment("Non-academic"))[Academic]	0.007	0.006	14715	10401	1
GYN	SDSC_binary	Colleague_num	0.004	0.003	13786	8826	1
GYN	SDSC_binary	C(Colleague_presence, Treatment("No"))[Yes]	0.009	0.008	13839	12240	1
GYN	SDSC_binary	C(Race_white, Treatment("Unchecked"))[Checked]	0.007	0.006	13981	10601	1
GYN	SDSC_binary	C(Treat_site_Gynecologic, Treatment("Unchecked"))[Checked]	0.009	0.008	14948	10891	1
GYN	SDSC_binary	1|Record_ID_sigma	0.008	0.006	5396	8252	1

Supplementary Table 16. Convergence parameters for the gynecologic case using binarized HD95 as the dependent variable.

Case	Metric	Variable	mcse_mean	mcse_sd	ess_bulk	ess_tail	r_hat
GYN	HD95_binary	Intercept	0.011	0.015	30804	20270	1
GYN	HD95_binary	C(ROI_type)[Tumor]	0.003	0.002	63090	26425	1
GYN	HD95_binary	C(Location)[US]	0.011	0.014	27641	20026	1
GYN	HD95_binary	C(Gender, Treatment("Male"))[Female]	0.006	0.005	28669	23373	1
GYN	HD95_binary	Total_years_of_practice	0.003	0.003	27968	20787	1
GYN	HD95_binary	C(Practice_type, Treatment("Non-academic"))[Academic]	0.006	0.006	25698	21841	1
GYN	HD95_binary	Colleague_num	0.003	0.002	28390	23149	1
GYN	HD95_binary	C(Colleague_presence, Treatment("No"))[Yes]	0.008	0.007	28357	22676	1
GYN	HD95_binary	C(Race_white, Treatment("Unchecked"))[Checked]	0.005	0.005	31844	24131	1
GYN	HD95_binary	C(Treat_site_Gynecologic, Treatment("Unchecked"))[Checked]	0.008	0.009	30527	22576	1
GYN	HD95_binary	1|Record_ID_sigma	0.008	0.006	7981	9819	1

Supplementary Table 17. Convergence parameters for the gastrointestinal case using binarized DSC as the dependent variable.

Case	Metric	Variable	mcse_mean	mcse_sd	ess_bulk	ess_tail	r_hat
GI	DSC_binary	Intercept	0.015	0.013	32625	22474	1
GI	DSC_binary	C(ROI_type)[Tumor]	0.004	0.003	54623	28418	1
GI	DSC_binary	C(Location)[US]	0.02	0.016	26614	22297	1
GI	DSC_binary	C(Gender, Treatment("Male"))[Female]	0.007	0.007	28502	21852	1
GI	DSC_binary	Total_years_of_practice	0.005	0.004	20796	21685	1
GI	DSC_binary	C(Practice_type, Treatment("Non-academic"))[Academic]	0.011	0.009	20980	21353	1
GI	DSC_binary	Colleague_num	0.007	0.005	22249	23536	1
GI	DSC_binary	C(Colleague_presence, Treatment("No"))[Yes]	0.008	0.007	31745	22070	1
GI	DSC_binary	C(Race_white, Treatment("Unchecked"))[Checked]	0.007	0.006	31258	23044	1
GI	DSC_binary	C(Treat_site_Gastrointestinal, Treatment("Unchecked"))[Checked]	0.013	0.01	28288	24776	1
GI	DSC_binary	1|Record_ID_sigma	0.006	0.004	13999	15694	1

Supplementary Table 18. Convergence parameters for the gastrointestinal case using binarized SDSC as the dependent variable.

Case	Metric	Variable	mcse_mean	mcse_sd	ess_bulk	ess_tail	r_hat
GI	SDSC_binary	Intercept	0.036	0.033	7032	5243	1
GI	SDSC_binary	C(ROI_type)[Tumor]	0.006	0.006	17715	14551	1
GI	SDSC_binary	C(Location)[US]	0.036	0.043	6230	5212	1
GI	SDSC_binary	C(Gender, Treatment("Male"))[Female]	0.016	0.014	7018	5575	1
GI	SDSC_binary	Total_years_of_practice	0.013	0.011	5192	4085	1
GI	SDSC_binary	C(Practice_type, Treatment("Non-academic"))[Academic]	0.024	0.017	6509	6451	1
GI	SDSC_binary	Colleague_num	0.017	0.018	5321	4717	1
GI	SDSC_binary	C(Colleague_presence, Treatment("No"))[Yes]	0.028	0.021	3998	3269	1
GI	SDSC_binary	C(Race_white, Treatment("Unchecked"))[Checked]	0.018	0.018	7561	5900	1
GI	SDSC_binary	C(Treat_site_Gastrointestinal, Treatment("Unchecked"))[Checked]	0.028	0.022	6095	4637	1
GI	SDSC_binary	1|Record_ID_sigma	0.022	0.021	3506	2309	1

Supplementary Table 19. Convergence parameters for the gastrointestinal case using binarized HD95 as the dependent variable.

Case	Metric	Variable	mcse_mean	mcse_sd	ess_bulk	ess_tail	r_hat
GI	HD95_binary	Intercept	0.049	0.063	7329	5593	1
GI	HD95_binary	C(ROI_type)[Tumor]	0.005	0.006	30731	23036	1
GI	HD95_binary	C(Location)[US]	0.029	0.021	9396	7166	1
GI	HD95_binary	C(Gender, Treatment("Male"))[Female]	0.02	0.018	8259	5344	1
GI	HD95_binary	Total_years_of_practice	0.012	0.011	8546	5023	1
GI	HD95_binary	C(Practice_type, Treatment("Non-academic"))[Academic]	0.028	0.02	7801	6558	1
GI	HD95_binary	Colleague_num	0.013	0.009	11423	11197	1
GI	HD95_binary	C(Colleague_presence, Treatment("No"))[Yes]	0.019	0.014	9849	9039	1
GI	HD95_binary	C(Race_white, Treatment("Unchecked"))[Checked]	0.021	0.016	7707	5448	1
GI	HD95_binary	C(Treat_site_Gastrointestinal, Treatment("Unchecked"))[Checked]	0.029	0.027	8642	6731	1
GI	HD95_binary	1|Record_ID_sigma	0.019	0.015	5485	3874	1
